# Interindividual differences in aronia juice tolerability linked to gut microbiome and metabolome changes—secondary analysis of a randomized placebo-controlled parallel intervention trial

**DOI:** 10.1186/s40168-024-01774-4

**Published:** 2024-03-09

**Authors:** Sonja Lackner, Alexander Mahnert, Christine Moissl-Eichinger, Tobias Madl, Hansjörg Habisch, Nathalie Meier-Allard, Christina Kumpitsch, Theresa Lahousen, Alexandra Kohlhammer-Dohr, Sabrina Mörkl, Herbert Strobl, Sandra Holasek

**Affiliations:** 1 Division of Immunology, Otto Loewi Research Center for Vascular Biology, Immunology and Inflammation, Medical University of Graz, Stiftingtalstraße 6, 8010 Graz, Austria; 2 Diagnostic and Research Institute of Hygiene, Microbiology and Environmental Medicine, Medical University of Graz, Neue Stiftingtalstraße 6, 8010 Graz, Austria; 3 Division of Medicinal Chemistry, Otto Loewi Research Center, Medical University of Graz, Neue Stiftingtalstraße 6, 8010 Graz, Austria; 4 BioTechMed-Graz, Mozartgasse 12/II, 8010 Graz, Austria; 5 Department of Psychiatry and Psychotherapeutic Medicine, Medical University of Graz, Auenbruggerplatz 31, 8036 Graz, Austria; 6 Division of Medical Psychology, Psychosomatics and Psychotherapeutic Medicine, Auenbruggerplatz 3, 8036 Graz, Austria

**Keywords:** Aronia juice, Polyphenols, Tolerability, Complaints, Mucus, Bile acid, Lipoprotein metabolism, Gut microbiome modeling, Metabolomics

## Abstract

**Background:**

*Aronia melanocarpa* is a berry rich in polyphenols known for health benefits. However, the bioavailability of polyphenols has been questioned, and the individual taste acceptance of the fruit with its specific flavor varies. We recently observed substantial differences in the tolerability of aronia juice among healthy females, with half of the individuals tolerating aronia juice without complaints. Given the importance of the gut microbiome in food digestion, we investigated in this secondary analysis of the randomized placebo-controlled parallel intervention study (ClinicalTrials.gov registration: NCT05432362) if aronia juice tolerability was associated with changes in intestinal microbiota and bacterial metabolites, seeking for potential mechanistic insights into the impact on aronia polyphenol tolerance and metabolic outcomes.

**Results:**

Forty females were enrolled for this 6-week trial, receiving either 100 ml natural aronia juice (verum, V) twice daily or a polyphenol-free placebo (P) with a similar nutritional profile, followed by a 6-week washout. Within V, individuals were categorized into those who tolerated the juice well (Vt) or reported complaints (Vc). The gut microbiome diversity, as analyzed by 16S rRNA gene-based next-generation sequencing, remained unaltered in Vc but changed significantly in Vt. A MICOM-based flux balance analysis revealed pronounced differences in the 40 most predictive metabolites post-intervention. In Vc carbon-dioxide, ammonium and nine O-glycans were predicted due to a shift in microbial composition, while in Vt six bile acids were the most likely microbiota-derived metabolites. NMR metabolomics of plasma confirmed increased lipoprotein subclasses (LDL, VLDL) post-intervention, reverting after wash out. Stool samples maintained a stable metabolic profile.

**Conclusion:**

In linking aronia polyphenol tolerance to gut microbiota-derived metabolites, our study explores adaptive processes affecting lipoprotein profiles during high polyphenol ingestion in Vt and examines effects on mucosal gut health in response to intolerance to high polyphenol intake in Vc. Our results underpin the importance of individualized hormetic dosing for beneficial polyphenol effects, demonstrate dynamic gut microbiome responses to aronia juice, and emphasize personalized responses in polyphenol interventions.

**Graphical Abstract:**

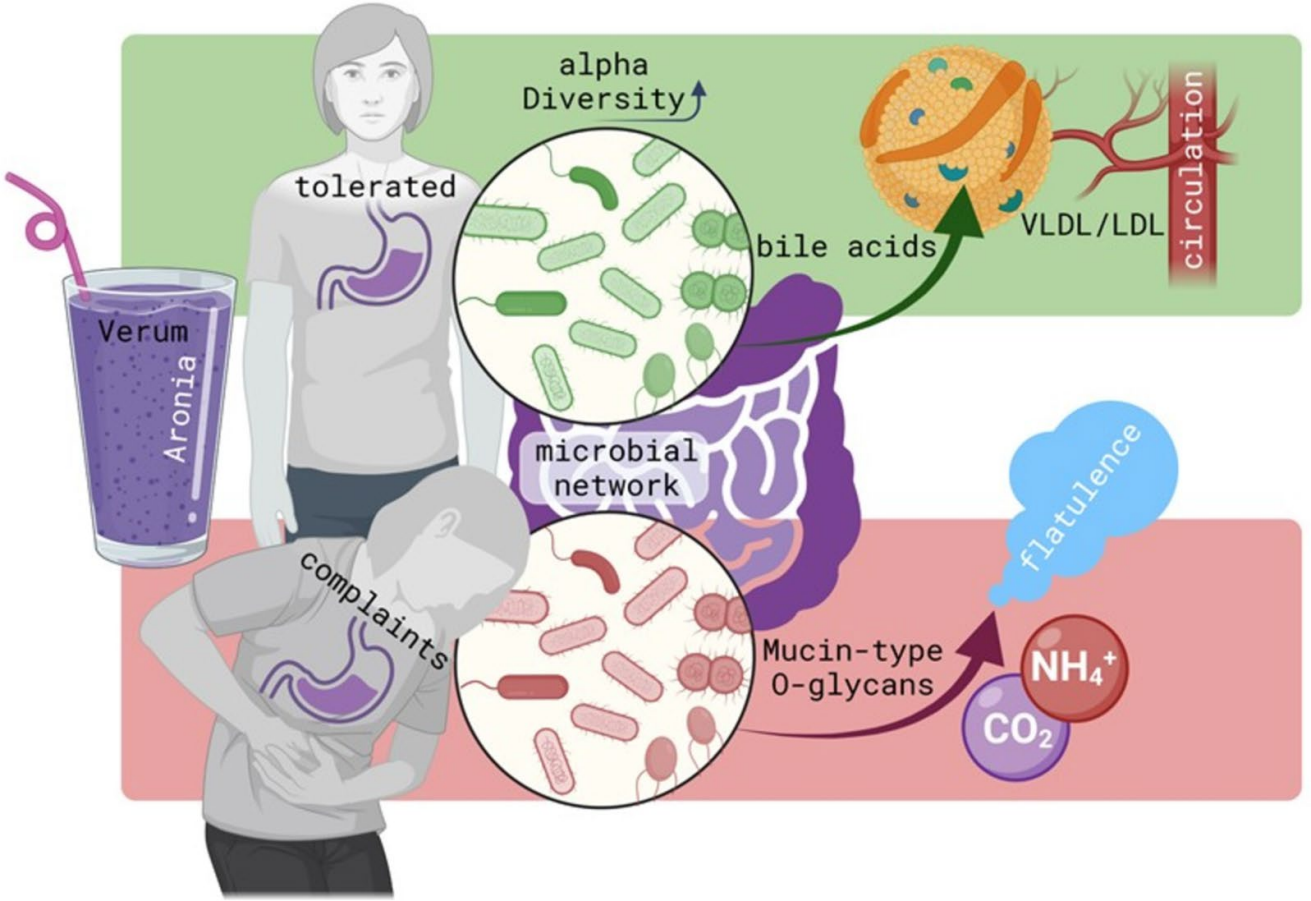

Video Abstract

**Supplementary Information:**

The online version contains supplementary material available at 10.1186/s40168-024-01774-4.

## Background

In recent years, *Aronia melanocarpa* (also known as chokeberry) has been promoted for its various beneficial health effects in humans. Thereby, many of the health-promoting effects of aronia fruits have been attributed to their high polyphenol content [1–3] which neutralizes free radicals via different mechanisms and have been reported to lead to a decrease of several biomarkers of metabolic and cardiovascular diseases [4, 5].

Polyphenols occur naturally in plant foods and fulfill several biological functions in plants [6, 7]. They contribute to attracting beneficial organisms to the plant through their coloring properties and they are part of the plants’ natural defense mechanisms against biological stressors [8]. Polyphenols are a large group of secondary plant metabolites with many hundreds of representatives. They are categorized into different subgroups such as flavonoids (such as flavonols, flavononols, flavones, flavanols, flavanones, anthocyanidins, isoflavonoids) and non-flavonoid (such as phenolic acid, stilbenes, coumarins, lignans, tannins) according to their chemical structure [9, 10].

In the human organism, dietary polyphenols have been reported to have a bidirectional beneficial interaction with the gut microbiota: On the one hand, they have been acknowledged to serve as prebiotics for certain gut microbes [11] by establishing symbiosis between the commensal gut microbes and the human organism. Polyphenols nourish symbiotic bacteria and promote their growth, thereby modulating microbial composition [12]. On the other hand, the metabolic utilization of polyphenols is determined by microbial cleavage of the complex polyphenolic structures. The absorption rate of polyphenols in the small intestine has been reported to be only 5–10% [13]. The great majority of polyphenols reach the colon where they are broken down and metabolized by gut microbes into smaller bioactive metabolites [14]. These metabolites are absorbed more efficiently and have higher bioavailability than their polyphenolic precursors [8]. Thus, the fermentation process in the colon enables the bioaccessibility of larger, complex polyphenols by providing smaller phenolic metabolites. Additionally, an ecological niche for bacterial species that trophically utilize these phenolic breakdown products is promoted. In parallel to prebiotic beneficial function, polyphenols inhibit the growth of pathogens in the gut, thereby contributing to eubiosis. These two opposed functions make them so-called duplibiotics [15].

Consequently, the physiological benefit of polyphenols may strongly be related to the individuals’ microbiome composition [3] which impacts the bioavailability and functionality of polyphenols in the human organism [12, 16, 17]. Furthermore, it may also be influenced by the individual dietary composition [18–20]. The bioactivity of polyphenols is also influenced by accompanying nutrients and food components like dietary fibers, carbohydrates, vitamins, and minerals that may influence the biotransformation of the phenolic compounds and their release from the food matrix to process them further [21].

Certain polyphenols have been reported to interact with the intestinal mucin-rich mucus layer, thus serving as an important factor for the mucus barrier function between the luminal microbiota and the intestinal wall with its underlying immune cells. The mucin-degrading capacity of the human microbiota is strongly influenced by nutritive factors [22, 23].

In *Aronia melanocarpa*, the predominant polyphenols are the flavonoids anthocyanins and procyanidins (which are condensed tannins that are formed from polymerization of the flavan-3-ols catechin and epicatechin [24]) as well as non-flavonoid phenolic acids (with chlorogenic acid and neochlorogenic acid as the main representatives that both are caffeic acids belonging to hydroxy-cinnamic acids) [25, 26]; however, the exact composition is influenced by growth conditions such as environmental and climate factors, such as temperature, sun exposure, and soil quality [27] as well as harvesting and storage conditions within the course of processing [28].

The majority of literature on aronia interventions describes beneficial health effects. However, despite the high polyphenolic content of aronia products the bioavailability of these components is described as being low [29] and may be dependent on personalized factors. Moreover, the acceptance of aronia products is inconsistent mainly due to the astringent, bitter, and sour taste which is attributed to the phenolic components such as tannins [30, 31]. Additionally, we recently described mixed tolerability of aronia juice and found a diverse response to an aronia juice intervention in a cohort of young healthy females [32]. Half of the participants tolerated the aronia juice well, whereas the other half reported gastrointestinal complaints after aronia juice consumption.

The pathophysiological mechanisms leading to adverse responses after aronia juice consumption are currently unknown. In this publication, we addressed the question of how aronia juice ingestion affects the host physiology and gut microbiome in healthy volunteers. Based on microbiome profiling, we used microbiome modeling and metabolite flux prediction to link microbiome activity with metabolome information from host gut and blood. We could show that according to the metabolite flux model the microbiome of volunteers that reported on complaints produced high quantities of gasses (carbon dioxide and ammonium) as well as several O-glycans after the juice consumption. In contrast, in volunteers that tolerated the juice several bile acids derived from microbial activity were predicted, which was most likely affirmed by a significant increase of several plasma lipoprotein subfractions (especially LDL- and VLDL) after the consumption of aronia juice.

## Methods

### Study design

#### Procedures and intervention

We conducted a to the participants single-blinded placebo-controlled human intervention study in parallel design to investigate the effects of aronia juice polyphenols in a healthy, normal weight, female cohort to assess potential alterations of the gut microbiome composition and the metabolic profile. Since the aronia juice was tolerated differently in the verum group [32], we aimed to identify a potential mechanistic contribution of the gut microbiome on the tolerability of the aronia juice.

**Fig. 1:**
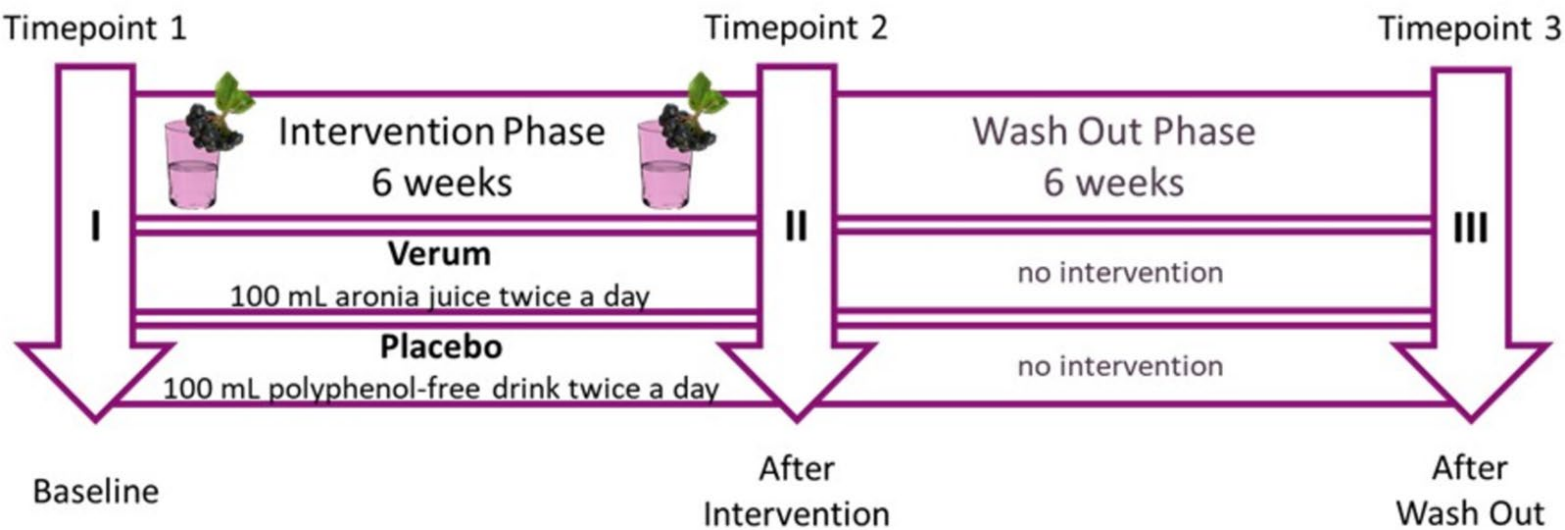
Study design. The study was performed over 12 weeks and divided into two phases: during the intervention phase of 6 weeks, the participants were asked to consume 100 mL of aronia juice or a polyphenol-free but similarly nutrient-composed placebo drink, respectively, twice a day. The participants were investigated at baseline (time point 1), after the intervention (time point 2), and after the washout phase (time point 3). The study was single-blinded to the participants and performed in parallel design

The study was conducted over 12 weeks and structured into a 6-week intervention and a 6-week wash-out phase. During the intervention phase, the participants were asked to consume 100 mL of locally produced, commercially available aronia juice (verum, V) or a placebo drink (P), respectively, twice a day amounting to a total volume of 200 mL daily. This amount has been chosen since previous studies testing drinks with high polyphenol content had been performed with this quantity [33–35]. As assessed by the Folin-Ciocalteu micro-method [36], the aronia juice polyphenol concentration was 8330 mg/L. The placebo drink was completely polyphenol-free and prepared according to a published recipe [37]. It was formulated using the average macro- and micronutrient composition of aronia juice and colored and flavored with polyphenol-free food dyes and flavorings, thus providing a comparable nutrient profile between the study drinks except for the polyphenol content. The drinks were provided in the same containers. Primary outcome parameter was defined as regulatory T cells as recently published [32]. Secondary outcome parameters focused on gut microbiome and the metabolome. The study participants were investigated at three time points: at baseline (I), after the intervention (II), and after the washout period (III) (Fig. 1). The examination took place at the investigation room of the Division of Immunology, Medical University of Graz.

The group assignment to V and P was randomized, single-blinded to the participants and stratified for age by applying a central computerized schedule (www.randomizer.at, provided by the Institute of Medical Informatics, Statistics and Documentation of the Medical University of Graz, Austria).

The ethical approval for this study was obtained by the Ethics Committee of the Medical University Graz (EK: 30-009 ex 17/18), the study was registered at ClinicalTrials.gov (NCT05432362), and the study was conducted in accordance with the Declaration of Helsinki. All participants gave their written informed consent and volunteered in this study. Data reporting followed the CONSORT 2010 Statement [38] including the considerations for nutritional trials [39].

#### Study population

In total, 40 normal-weight females aged between 18 and 40 years were recruited by the principal investigator for this study in February 2019 via the local universities, libraries, sports clubs, and word-of-mouth advertisement. Since aronia juice is known for its bitter and astringent taste not everyone favors, the participants received information on the specific taste of the study drinks in advance to select individuals who could imagine consuming this specific drink for several weeks. The inclusion criteria for enrollment were as follows: female sex, age between 18 and 40 years, preference for astringent flavors, and absence of exclusion criteria. The following exclusion criteria were applied: known fructose malabsorption and fructose intolerance, acute diseases within the previous 2 months or chronic diseases or infections (including upper respiratory tract infections, fever, chronic inflammatory disorders, autoimmune-disorders), a history of digestive diseases such as inflammatory bowel disease and irritable bowel syndrome, history of gastrointestinal surgery other than appendectomy, pregnancy, and period of breastfeeding, as well as antibiotic or antifungal treatment within the previous 2 months and daily or irregular intake of supplemental prebiotics or probiotics within the previous 2 months except for natural probiotic food such as yogurt and dairy products. Details on the enrollment procedure are depicted in the CONSORT diagram (Additional file 1).

#### Lifestyle monitoring

The participants were instructed to follow their usual diet and maintain their lifestyle behaviors and to avoid intentional weight changes during the study participation to keep the general metabolic conditions largely constant. Anthropometric measures such as body height, body weight, and waist circumference were measured at all three time points, and the body mass index (BMI) was calculated according to the formula BMI = body weight [kg]/body height [m]^2^ [40]. Information on smoking behavior was collected by the Fagerström test for nicotine dependency [41]. To assess nutritional key data, the participants were asked to document their food intake for four days prior to the three investigation appointments. The Vienna Food Record [42] was applied which takes Austrian-specific eating habits into account. The nutrient intake was analyzed by the Austrian specific nutritional software nut.s ® v1.32.95 (www.nutritional-software.at, dato Denkwerkzeuge, Vienna Austria).

#### Compliance assessment, complaint questionnaire, and tolerability groups

To keep the compliance and motivation of the participants high and to identify any issues at an early stage, the participants were contacted regularly via phone calls and e-mails. The returned drink containers were weighted at investigation II after the intervention to control for compliance. Additionally, the participants were asked to complete a complaints and preference questionnaire (adopted from [43]) to assess the tolerability of the study drinks. As reported in Lackner et al. [32], half of the participants reported clear gastrointestinal complaints like nausea, diarrhea, obstipation, bloating, and cramps. Thus, V was further divided into the group that tolerated the aronia juice well (Vt) and the group that reported clear complaints (Vc) associated with the juice ingestion.

#### Blood sample collection

Blood draws were performed in overnight fasted participants with food fasting of at least 12 h. Participants were allowed to drink tap water before the blood draw. Plasma and serum samples were centrifuged at 3000g for 10 min. Three hundred microliters of aliquots was prepared and stored at – 80 °C. For further analyses, the samples were thawed at room temperature and centrifuged for 5 min at 10,000g. Further sample preparation procedures are described in detail at the metabolomics section.

### Gut microbiome

#### Sample collection, DNA extraction and amplicon sequencing

Approximately 1 g of stool was collected from each participant using stool sample containers (Meus S.R.L., Piove di Sacco, Italy) and stored at – 20 °C until further processing. The microbiome sequencing protocol, based on Illumina Miseq sequencing, was described earlier by Klymiuk et al. [44]. The protocol for these samples’ preparation deviated in minor ways, like using 250 μl of Bacterial Lysis Buffer in the MagNA Lyser green beads tubes (Roche, Mannheim, Germany), adding 250 μl of a stool–PBS buffer suspension to it, and homogenizing it two times 30 s at 6500 rpm with the MagNA Lyser Instrument (Roche, Mannheim, Germany), instead of three times. For the incubation step with Proteinase K (20 mg/ml), 1 h sufficed. The Magna Pure LC DNA III Isolation Kit (Bacteria, Fungi) (Roche, Mannheim, Germany) was used to extract DNA according to the manufacturer’s instructions. The hypervariable V4 region of the bacterial 16S rRNA gene was amplified with polymerase-chain-reaction (PCR) from the fecal total DNA using the target-specific primers 515fModified – GTGYCAGCMGCCGCGGTAA [45] and 806rModified – GGACTACNVGGGTWTCTAAT [46]. Two microliters of the total DNA was used in a 25-μL PCR reaction in triplicates with the FastStart High Fidelity PCR system, dNTPack (Sigma, Germany). Cycling conditions were of initial denaturation at 95 °C for 3 min, followed by 30 cycles of 95 °C for 45 s, 55 °C for 45 s, 72 °C for 1 min, and a final elongation step at 72 °C for 7 min. The resulting amplification products were visualized on a 1.5% agarose gel and pooled, indexed and purified as described in Klymiuk et al. [44], with the difference that 7.5 μl normalized PCR product was used in a 25-μl indexing PCR reaction; 9 pM of the final library were sequenced at ZMF Core Facility Molecular Biology in Graz, Austria, using an Illumina MiSeq desktop sequencer with v3 chemistry and 600 cycles (2 × 300). FASTQ files were used for data analysis.

#### Sequence data processing

Raw reads were analyzed with QIIME2 (Quantitative Insights Into Microbial Ecology) version 2021.4 using DADA2 (Divisive Amplicon Denoising Algorithm) to denoise sequences [47, 48]. Briefly, paired-end reads were joined together before a quality check of the produced sequences was performed. Afterward, the taxonomic assignment was realized with a Naïve-Bayes classifier trained on the Silva138 reference database [49].

#### Controls and decontam

Extraction blanks and PCR negative controls were processed in parallel. Contaminating reads were removed using the R package decontam [50] with the prevalence method and threshold set to 0.5 (https://github.com/benjjneb/decontam).

#### Data normalization

Data were normalized to 6000 reads/ sample using R package SRS (Scaling with ranked subsampling) [51].

#### Differential abundance analysis and visualization of microbiome data

Analyses and visualization were performed using RStudio, based on R version 4.1.2 [52]. Package ALDEx2 [53] was used for differential relative count abundance analysis for the comparison of two conditions. Used R scripts are provided in the GitHub repository (https://github.com/Christine-Moissl-Eichinger/aronia). Final figures were compiled using Inkscape [54].

#### Metabolic predictions

Potential metabolites were predicted with the q2-micom plugin (v. 0.12.1) [55]. All analyses were conducted with the AGORA genus model database (v1.03) [56] and the standard western diet gut medium. The default abundance cutoff (0.0001) was used. The growth simulation was performed with individual settings for the tradeoff between community growth rate and individual taxon growth rate. This pressure on the model was determined by an evaluation of the tradeoff from 0 to 1 (zero to maximum enforced growth) and was set at 0.1 and 0.2 respectively. Subsequent visualizations and analysis included potential metabolite consumptions, growth niches, and metabolite fluxes in dependence on Vc and Vt for all and individual time points.

### Metabolome stool and plasma

#### Metabolic quantification using NMR

Nuclear magnetic resonance spectroscopy (NMR) analysis was used to analyze concentrations of acetate, succinate, formate, lactate, butyrate, and propionate in stool samples (PMA untreated) performed at the Gottfried Schatz Research Center for Cell Signaling, Metabolism and Aging, Molecular Biology and Biochemistry, Medical University of Graz. To quench enzymatic reactions and remove proteins, methanol-water solution was added to the stool samples (2:1), and cells were lysed using a Precellys homogenizer and stored at – 20 °C for 1 h until further processing. Samples were centrifuged (4 °C, 30 min, 17949 rcf), and supernatants were lyophilized afterwards.

Samples were then mixed with 500 μl NMR buffer in D_2_O (0.08 M Na_2_HPO_4_, 5 mM 3-(trimethylsilyl) propionic acid-2,2,3,3-d_4_ sodium salt (TSP), 0.04 (w/v) % NaN_3_, pH adjusted to 7.4 with 8 M HCl and 5 M NaOH) and transferred into 5-mm NMR tubes. NMR measurements were performed on an AVANCE™ Neo Bruker Ultrashield 600 MHz spectrometer equipped with a TXI probe head at 310 K and processed as described elsewhere [57].

The 1D CPMG (Carr-Purcell_Meiboom_Gill) pulse sequence (cpmgpr1d, 128 scans, 73728 points in F1, 11904.76 HZ spectral width, recycle delays 4 s) with water suppression using pre-saturation was used for ^1^H 1D NMR experiments. Bruker Topspin version 4.0.2 was used for NMR data acquisition. The spectra for all samples were automatically processed (exponential line broadening of 0.3 Hz), phased and referenced using TSP at 0.0 ppm using the Bruker Topspin 4.0.2 software (Bruker GmbH, Rheinstetten, Germany).

Spectra pre-processing and data analysis have been carried out using the state-of-the-art data analysis pipeline (group of Prof. Jeremy Nicholson at Imperials College London) using Matlab® scripts and MetaboAnalyst 4.0 [58]. NMR data were imported to Matlab® vR2014a (Mathworks, Natick, Massachusetts, USA), regions around the water, TSP, and remaining methanol signals excluded, to correct for sample metabolite dilution probabilistic quotient normalization [59] as performed.

Blood plasma low molecular weight metabolites and lipoproteins were analyzed on a Bruker 600 MHz Avance Neo NMR spectrometer (Bruker, Rheinstetten, Germany). Among the measured analytes (in total 41) are amino acids and their derivatives, carboxylic acids, ketone bodies, and monosaccharides. Plasma samples were thawed, and 330 μl of each sample was mixed with 330 μl of Bruker plasma buffer (Bruker, Rheinstetten, Germany). Following gentle mixing, 600 μl of the samples were transferred into 5 mm glass tubes and placed into a SampleJet rack (Bruker, Rheinstetten, Germany). Proton spectra were obtained at a constant temperature of 310 K using a standard nuclear Overhauser effect spectroscopy (NOESY) pulse sequence (Bruker: noesygppr1d), a Carr–Purcell Meiboom–Gill (CPMG) pulse sequence with pre-saturation during the relaxation delay (Bruker: cpmgpr1d) to achieve water suppression, and a fast scan 2D J-resolved (JRES) pulse sequence (Bruker: jresgpprqf). Data analysis was carried out using the Bruker IVDr Plasma (B.I.) module of the analysis software (Topspin version 4.1). Univariate statistical analysis of the concentration values for identifying lipoproteins and metabolites of interest differing between groups was carried out using MetaboAnalyst [60] and the R package rstatix.

### Statistical analysis

At the conceptual phase of this study, no previous randomized controlled trial on the effects of dietary polyphenols or aronia food products on the primary outcome were available. Consequently, we were unable to determine the effect size. To detect an effect of 1 standard deviation at a significance level of 0.05 and 80% power, a minimum of 16 individuals per group is required. Therefore, we opted for a sample size of *n* = 40, with 20 participants per group. Comparable studies employing similar interventions but focusing on different primary outcomes had sample sizes ranging from 20 to 66 individuals [33, 61, 62]. Moreover, we conducted effect size and power calculations with q2-evident (https://github.com/biocore/evident) on the microbiome data and the predicted flux of metabolites in our study (Additional file 2).

Alpha diversity estimates were compared with t-tests for paired and normally distributed samples. Differential relative count abundance analysis was based on ALDEx2, Wilcoxon rank tests, and Benjamin Hochberg for multiple comparison corrections. L1 penalized logistic regression was used to identify the most predictive metabolites for groups complaints vs. tolerated. Two- and three-way ANOVA analyses were applied to detect significant differences of measured metabolites in plasma, stool, and lipoproteins.

### Data and software availability

Raw sequencing data obtained from amplicon-based sequencing (technical sequences including adaptor sequences, linker sequences and barcode sequences were removed) used in this paper can be found in the European Nucleotide Archive (ENA) und the accession number PRJEB64786 (https://www.ebi.ac.uk/ena/browser/home). Datasets (abundance table, taxonomy table, etc.) are available at GitHub: https://github.com/Christine-Moissl-Eichinger/aronia.

The NMR raw data has been deposited at MetaboLights under the accession number MTBLS7661 (https://www.ebi.ac.uk/metabolights/). *Comment: Until the data release on MetaboLights, the data is provided via nexcloud and can be accessed at the following links: Stool data: *https://box.medunigraz.at/s/NAPq6E9mTbPmF5L*, plasma data: *https://box.medunigraz.at/s/zomSiTNkafkXRDY.

## Results

### Study population characteristics and tolerability groups

In total, 40 female participants were included in this study and randomly allocated to the verum/aronia juice (V) and the placebo (P) group with 20 participants each (Table 1). One participant of V and two participants of P dropped out at the first follow-up investigation after the intervention phase, and another two participants of V dropped out at the final investigation after the washout period (Additional file 1).

**Table 1:** Study population characteristics at baseline. Data are presented as median (IQR). *p*-values were derived from Mann-Whitney *U* test if not marked, from Fisher’s exact test if marked with Ɨ, and from chi-square test if marked with Ɨ Ɨ

**Study population characteristics**	**Verum**	**Placebo**	***p*-value**
Number of participants (*n*)	20	20	
Age (years)	25 (7)	24 (5)	0.142
Smokers	4	3	1.000^Ɨ^
BMI (kg/m^2^)	21.2 (2.9)	21.6 (3.2)	0.841
Waist circumference (cm)	68.3 (9.3)	68.5 (4.8)	0.758
Drop out before first follow up	1	2	0.307^Ɨ Ɨ^
Drop out before second follow up	2	0	
**Study drink tolerability**			
Tolerated (*n*)	9	17	0.003^Ɨ^
Complaints (*n*)	10	1	
**Characteristics tolerability groups**	**Verum tolerated**	**Verum complaints**	
Number of participants (*n*)	9	10	
Age (years)	29 (12)	24.5 (4)	0.133
Smokers	2	2	1.000^Ɨ^
BMI (kg/m^2^)	22.0 (2.5)	20.1 (3.9)	0.043
Waist circumference (cm)	73 (9.0)	66.3 (5.4)	0.022
**Dietary intakes**			
Energy (kcal/day)	1875 (724)	2054 (883)	0.211
Protein (g/d)	63 (26)	79 (18)	0.065
Carbohydrates (g/d)	182 (90)	210 (134)	0.133
Sugar (g/d)	75 (41)	91 (62)	0.447
Fat (g/d)	71 (43)	82 (38)	0.905
Saturated fat (g/d)	25 (19)	29 (14)	0.842
Fibers (g/d)	23 (15)	23 (31)	1.000
Polyphenols (mg/day)	450 (925)	644 (357)	0.780

Within V, nine participants reported having tolerated the juice well (Vt), and 10 participants reported gastrointestinal discomfort and complaints (Vc) such as bloating, stomach ache, nausea, and diarrhea, whereas the participants of P tolerated the placebo drink with one exception [32]. Based on this information, the tolerability groups have been considered for further statistics. Metadata information on the study population (group allocation, age, BMI, smoking and nicotine dependency at baseline, and weight, waist circumference and key nutritional information of all three measurement points) is provided in Additional file 3.

All participants provided stool and plasma samples at all three measurement points and completed the nutritional questionnaires. Thereof, the following data sets were obtained: “universal” 16S rRNA gene profiles for all stool samples, as well as metabolomic information of stool and plasma, and detailed dietary information (e.g., diversity, energy, protein, fat, carbohydrates) (Additional file 3).

### Gut microbiome composition

#### Shannon index increased significantly in the verum group over time

**Fig. 2:**
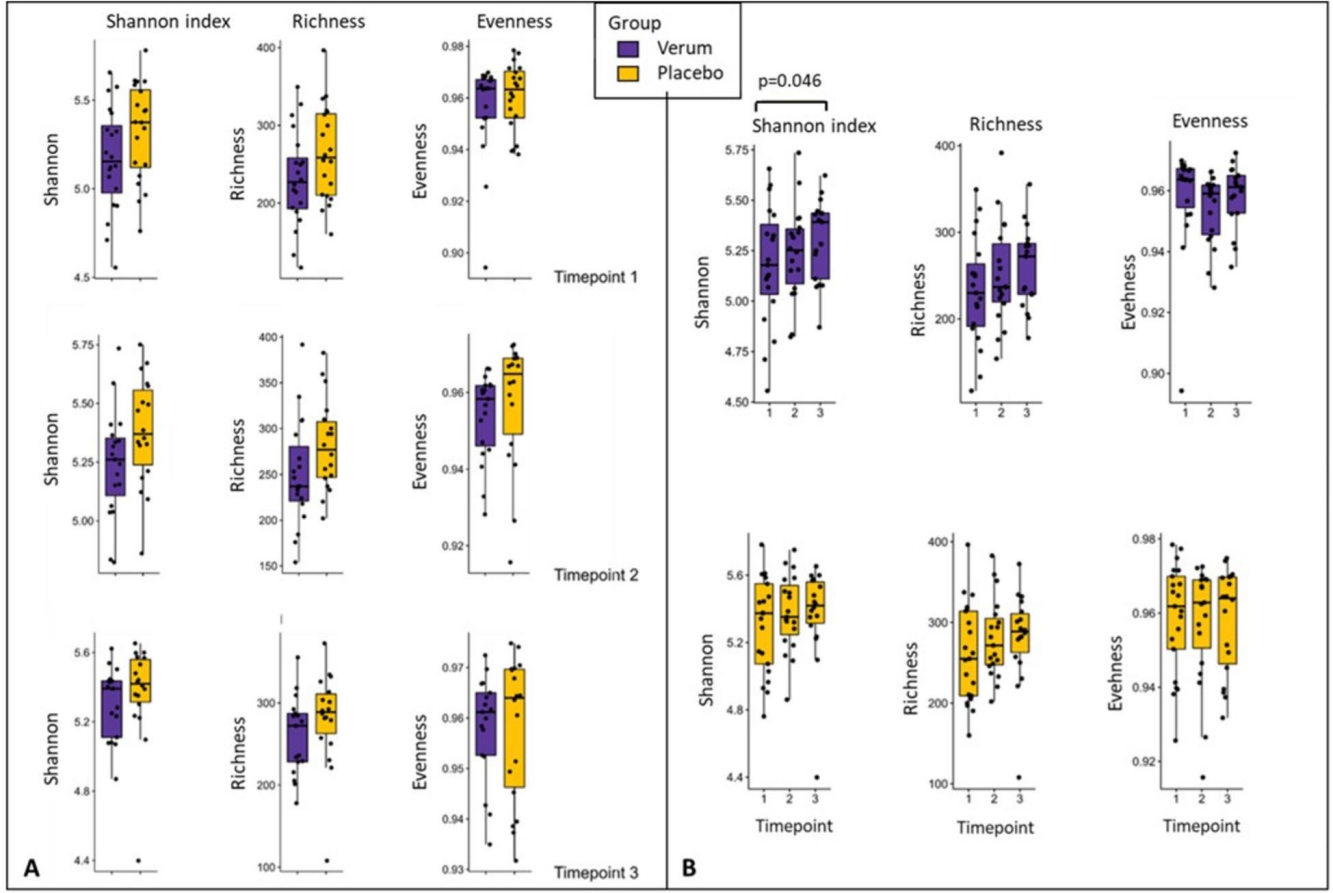
Differences in alpha diversity between the verum (V) and the placebo (P) group. The data is based on 16S rRNA gene amplicon sequencing and results are shown for the whole study population of *n* = 40. **A** Diversity indicators of V and P did not differ significantly between V and P at the three time points (1: at baseline, 2: after the intervention, 3: after the wash-out period). **B** Progression of diversity within the groups. In V, Shannon index increased continuously (significant difference as indicated by given *p*-value from time point 1 to time point 3, based on *t*-test, paired samples), while it remained constant in P

The diversity indices (Shannon index, richness, and evenness) did not differ between V and P at the three individual time points (Fig. 2A). However, alpha diversity increased continuously in V from time point 1 to 3 (*p* = 0.046; normal distribution, *t*-test for paired samples), while it remained constant in P (Fig. 2B; all datasets and scripts available in GitHub repository, see Materials and Methods).

#### Focusing on tolerability groups, alpha diversity, and richness increased continuously in the verum-tolerated group over time while it did not change in the complaints group

**Fig. 3:**
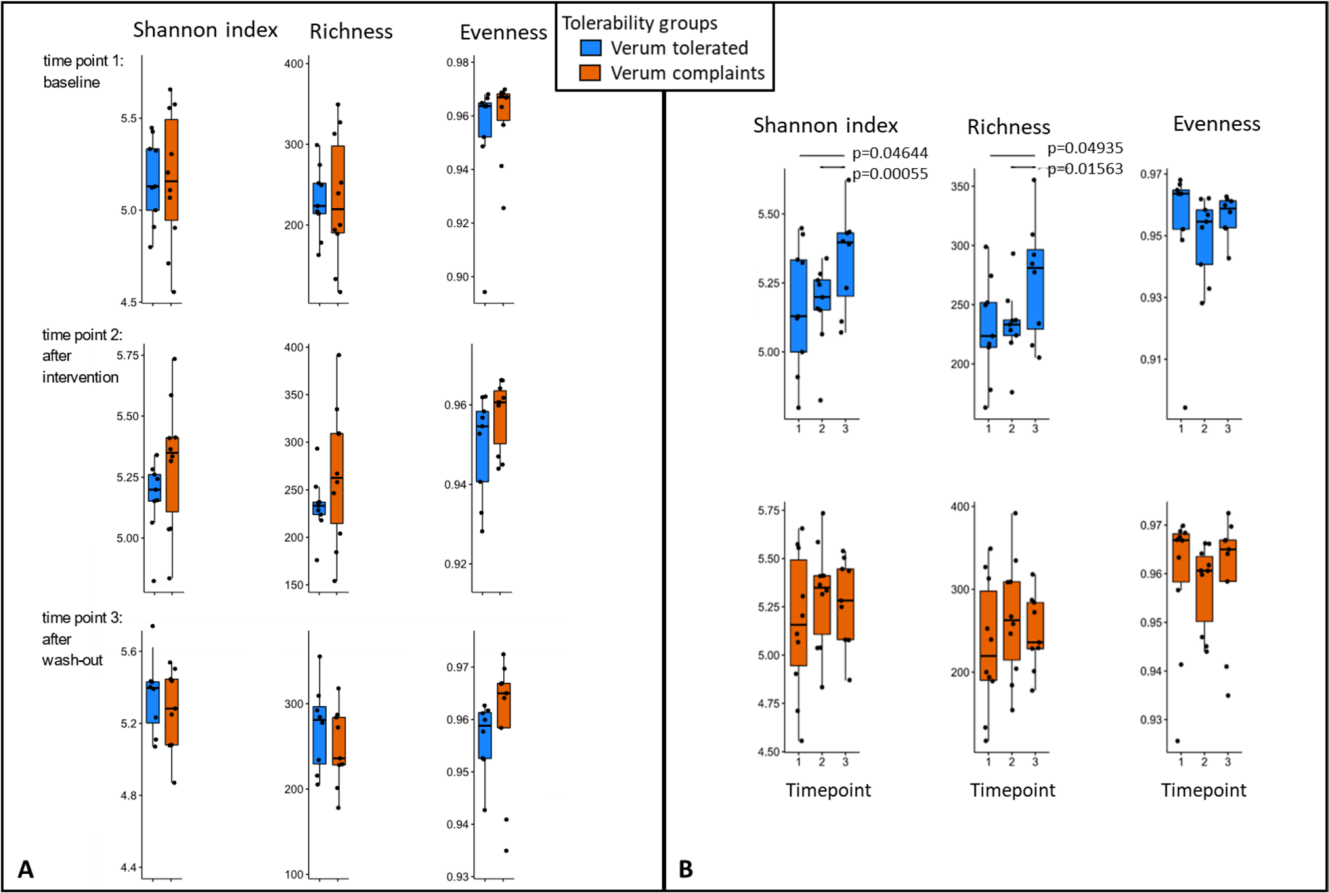
Differences in alpha diversity between the verum tolerated (Vt) and the verum complaints (Vc) group. The data is based on 16S rRNA gene amplicon sequencing and results are shown for the verum group of *n* = 20. **A** Diversity indicators did not differ significantly between Vt and Vc at the three time points (1: at baseline, 2: after the intervention, 3: after the wash-out period). **B** In Vt, the Shannon index and richness increased significantly over the study period (normal distribution, *t*-test for paired samples), while no significant differences were observed for Vc

To understand the effect of the juice and its correlation with tolerability, we separated the groups for tolerance (Vt) and complaints (Vc) after juice consumption. Although the two groups were not significantly different concerning alpha-diversity measures at all time points, the Vt group showed a significant increase in Shannon index and richness towards time point 3, while no changes were observed for Vc (Fig. 3, all significant *p*-values included; all datasets and scripts available in GitHub repository, see Materials and Methods). This observation indicates a potential adaptation by the gastrointestinal microbiome towards the aronia juice.

#### The genera Anaerostipes and Bacteroides showed an increasing trend in Vt

**Fig. 4:**
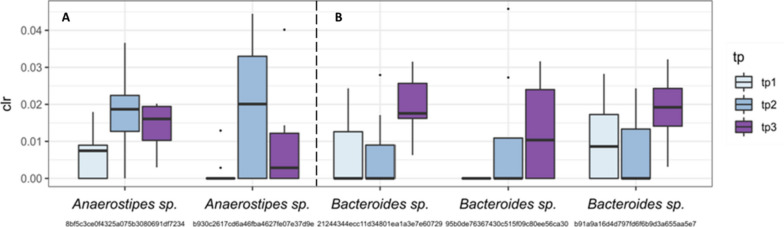
Changes in Vt group only. Species of the genera *Anaerostipes* and *Bacteroides* showed an increasing trend in the Vt group (*n* = 9). **A** *Anaerostipes* increased after the intervention and dropped again in the wash-out period. **B** *Bacteroides* started to increase after juice consumption. The changes did not remain significant after statistical Benjamini-Hochberg correction for multiple testing, and therefore *p*-values are not indicated. tp, time point; RSVs, ribosomal sequence variants. Feature ID for the RSVs is given below the genus information

To further understand the potential adaptation of the gut microbiome, and the possibly involved taxa, we performed differential relative count abundance analysis using ALDEx2 of the Vt group samples. Ribosomal sequence variants (RSVs) affiliated with the genus *Anaerostipes* raised from time point 1 to 2 and dropped again after the washout period (Fig. 4A; RSV 8bf[…], tp 1:2 *p* = 0.03, tp 1:3 *p* = 0.068, tp 2:3 *p* = 0.470; RSV b93[…], tp 1:2 *p* =0.142, tp 1:3 *p* = 0.385, tp 2:3 *p* = 0.480; all: Wilcoxon rank test, ALDEx2), while a constant increase toward time points 2 and 3 was observed for representatives of the genus *Bacteroides* (Fig. 4B; RSV 212[…], tp 1:2 *p* = 0.730, tp 1:3 *p* = 0.042, tp 2:3 *p* = 0.028; RSV 95b[…], tp1:2 *p* = 0.186, tp 1:3 *p* = 0.056; tp 2:3 *p* = 0.609; RSV b91[…], tp1:2 *p* = 0.713, tp 1:3 *p* = 0.140, tp 2:3 *p* = 0.058; Wilcoxon rank test, ALDEx2; all datasets and scripts available in GitHub repository, see Materials and Methods). Of note, observed changes did not remain significant after correction after statistical Benjamini-Hochberg correction for multiple testing.

At genus level, an increase from tp 1:2 was observed for *Ruminococcus* (*p* = 0.005), for 1:3 for [Eubacterium]_coprostanoligenes_group (*p* = 0.01), Lachnospiraceae_UCG-004 (*p* = 0.04), and for 2:3 for *Odoribacter* (*p* = 0.009; all: Wilcoxon rank test, ALDEx2; all datasets and scripts available in GitHub repository, see Materials and Methods). Again, after correction for multiple testing, none of these remained significant.

#### MICOM model-based flux balance analysis of keystone taxa revealed ammonium, CO_2_, and several O-glycans as likely metabolites in Vc after the juice consumption and several bile acids in Vt

**Fig. 5:**
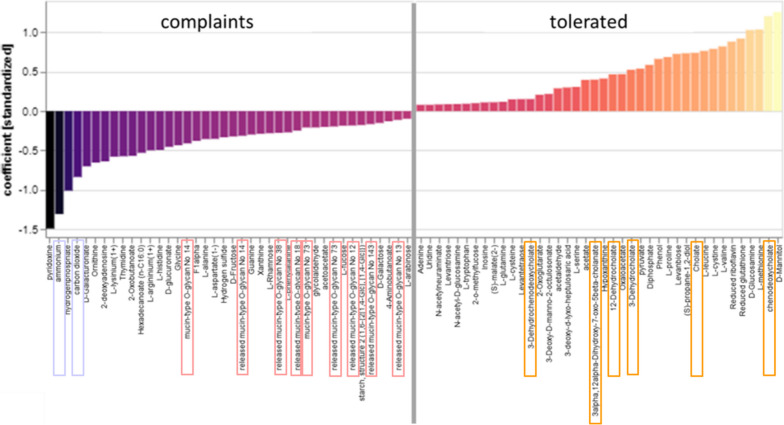
MICOM model-based flux balance analysis of keystone taxa at time point 2. The 40 most predictive production fluxes (metabolites) are shown for the verum subgroup that had complaints after aronia juice consumption (Vc) on the left and the verum subgroup that tolerated the juice well (Vt) on the right using L1 penalized logistic regression. The analysis was based on 16S rRNA gene amplicons and the identified keystone taxa of the samples collected at the time point 2 which was directly after the intervention. In Vc, ammonium and carbon dioxide are highlighted in violet, and O-glycans are highlighted in red. In Vt, bile acids and bile acid anions are highlighted in orange

The MICOM model-based flux balance analysis of keystone taxa revealed striking differences between the predictive production of metabolites from gut microbiota in Vc and Vt at time point 2 after the juice intervention. Of note, almost no differences in flux analysis were found between these two groups at baseline and at time point 3, respectively. Strikingly, in Vc, ammonium and carbon dioxide were among the five most likely metabolites after the juice consumption, and nine of the 40 most frequently predicted metabolites in Vc were O-glycans, while no O-glycans were to be expected in Vt. On the contrary, in Vt, six of the top 40 metabolites were bile acids and bile acid anions, respectively, thus indicating a connection to cholesterol metabolism, while none of these metabolites were present in Vc (Fig. 5).

**Fig. 6:**
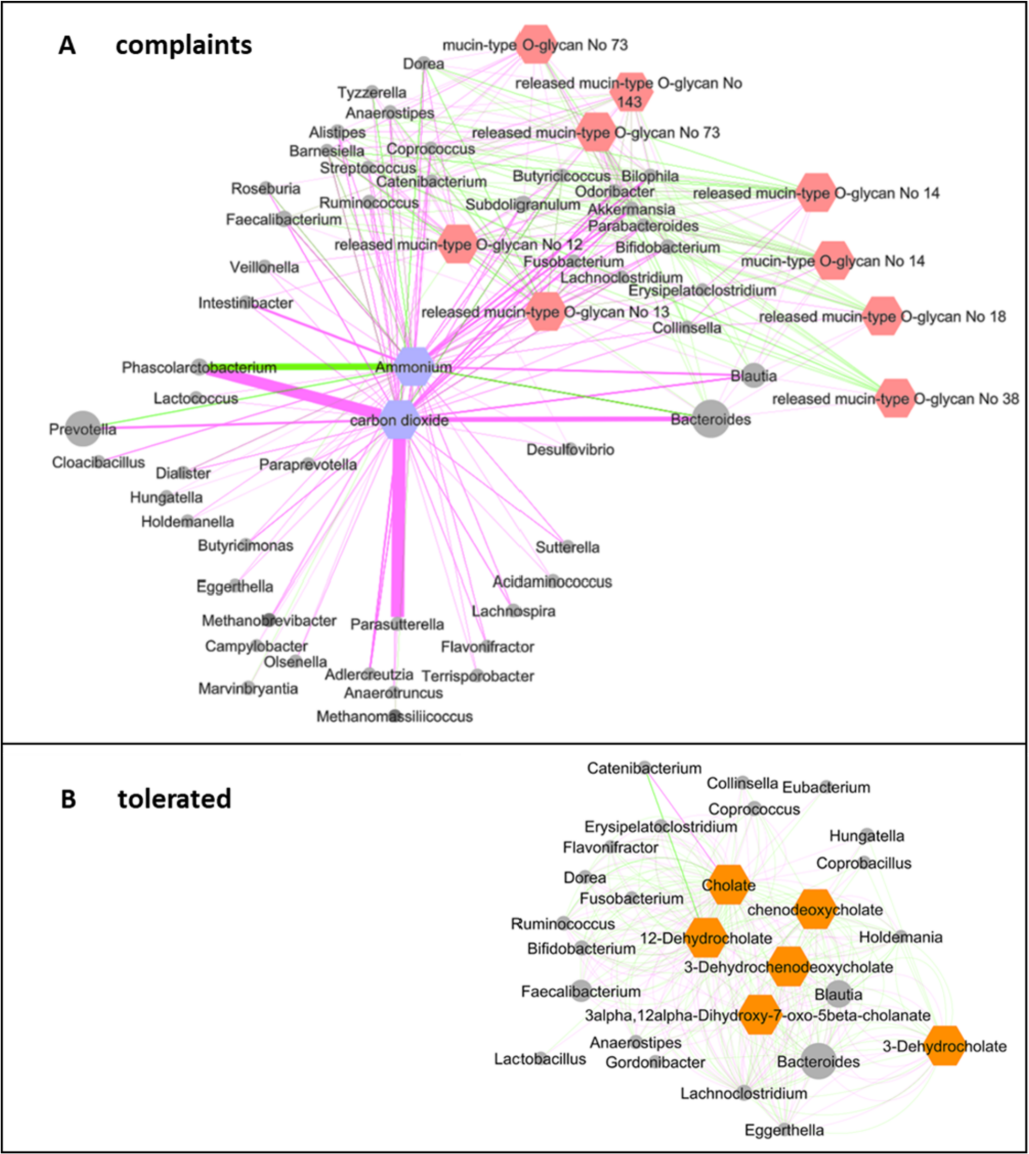
Networks of taxa and selected metabolites for verum complaints and verum tolerated at time point 2. The metabolites have been chosen in accordance with Fig. 5. **A** In Vc (complaints), ammonium and carbon dioxide are highlighted in violet, and O-glycans are highlighted in rose. **B** In Vt (tolerated), the predicted bile acids are highlighted in orange. In both networks, node size (metabolites and taxa) was scaled according to abundance; bacteria are highlighted in light grey and archaea (methanogens) in dark grey; edges were weighted according to their modeled flux, imported metabolites are connected by green lines, and exported metabolites are connected by pink lines. Network layout is based on an edge-weighted spring-embedded algorithm based on metabolic flux

As a next step, we had a closer look at these specific metabolites and extracted all taxa which could have been involved in their production or consumption flux. This data was further visualized as a comparative metabolic network of complaints (Vc) vs. tolerated (Vt) after aronia juice intervention (time point 2) (Fig. 6). Both networks differed according to their import and export flux.

In Vc, ammonium and carbon dioxide accumulated due to missing consumers and led to an unbalanced flux. The export—in other words the production—of carbon dioxide was predominantly driven by *Phascolarctobacterium*, *Parasutterella*, and *Bacteroides* as well as *Prevotella* and *Blautia*. For the export of ammonium, also *Blautia* appeared to be a relevant driver; however, for the occurrence of both fermentation products, a complex network of microbes was involved. The import—which means the consumption of substances by microbes—of ammonium was mainly related to *Phascolarctobacterium* and *Bacteroides*, but still consumers were overall underrepresented (Fig. 6A).

The network from Vt suggests a balanced and robust metabolic network of bile acids (Fig. 6B). The abundance of the taxa *Anaerostipes* and *Bacteroides* changed during the study in Vt (see Fig. 4) and contributed to the network of bile acid occurrence. Besides *Anaerostipes* and *Bacteroides*, *Blautia*,* Faecalibacterium*, and others were involved in the network of Vt.

In both Vc and Vt, the taxa *Anaerostipes* and *Bacteroides* played a role in the network. They occurred at divergent positions in the network for the Vc: *Bacteroides* was mainly involved in ammonium consumption and carbon dioxide release, while *Anaerostipes* was stronger connected to the metabolism of O-glycans. In contrast, in Vt *Anaerostipes* and *Bacteroides* were embedded in very similar metabolic networks. This adopted microbial community network could be linked to a better tolerability of aronia juice consumption.

### NMR analysis of plasma samples reveals changes in small-molecule and lipoprotein metabolism

Given the partially contradictory results on the metabolic impact of aronia juice on lipoprotein metabolism in humans [63, 64], we carried out a comprehensive NMR-based metabolomics analysis of plasma lipoproteins and metabolites of the 111 plasma samples obtained in this study using NMR spectroscopy.

**Fig. 7:**
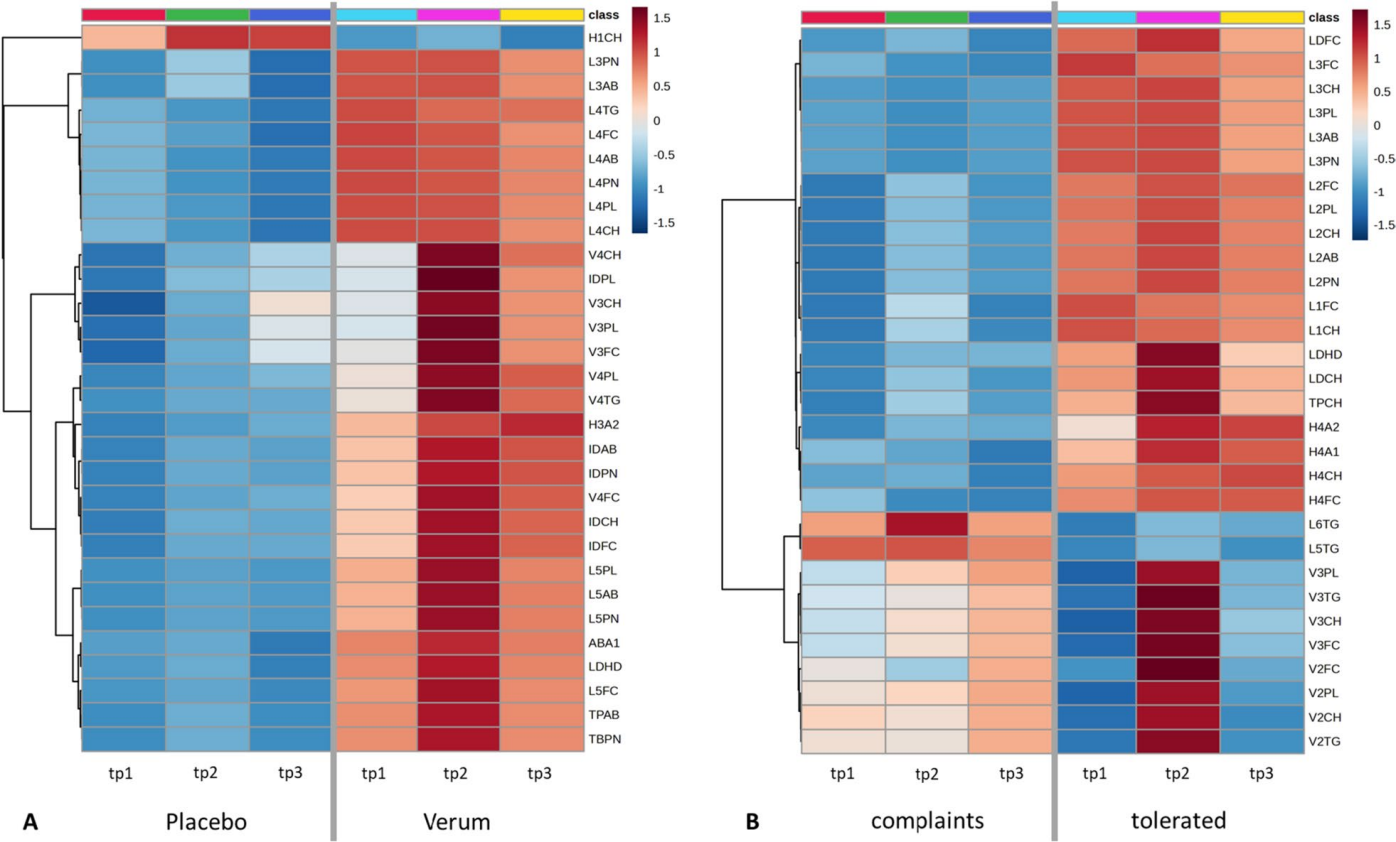
Changes in plasma lipoprotein profile over the study period. The 30 most abundant plasma lipoproteins are shown. **A** The change in plasma lipoprotein profile between the placebo and the verum group is depicted. In the placebo group plasma, lipoproteins were lower compared to the verum group. In the verum group, several plasma lipoproteins increased during the intervention with aronia juice (tp 2), whereas the strongest effects were observed for VLDL, IDL, and LDL sub-fractions. After the intervention (tp2), more than 30 lipoproteins were significantly higher in V compared to P. After the washout phase (tp 3), most of these altered lipoprotein plasma concentrations returned to initial concentrations except for ABA1, IDAB, IDCH, IDFC, and IDPN that remained significantly higher compared to baseline concentrations (tp 1). **B** The change in plasma lipoprotein profile in the tolerability groups during the intervention is depicted. A significant increase in plasma LDL-lipoproteins was only observed in the group that tolerated the aronia juice. The concentration of most lipoproteins returned to initial levels after the wash out phase. P, placebo; Tp1, time point 1 (baseline); Tp2, time point 2 (after the intervention); Tp3, time point 3 (after the washout phase); V, verum

Although we detected no significant differences in the plasma metabolite profiles between the placebo and verum groups after the intervention, we detected strong changes in lipoprotein profiles after the intervention (Additional file 4). More than 30 parameters associated with lipoprotein metabolism were significantly higher in V after the intervention compared to P (ABA1, H3A2, IDAB, IDCH, IDFC, IDPL, IDPN, L4AB, L4CH, L4FC, L4PL, L4PN, L4TG, L5AB, L5CH, L5FC, L5PL, L5PN, LDHD, TBPN, TPAB, V3CH, V3FC, V3PL, V3TG, V4CH, V4FC, V4PL, V4TG, VLAB, VLCH, VLPN). We observed the strongest differences in lipids and cholesterol species bound to VLDL, IDL and LDL sub-fractions. Of note, 5 lipoprotein-related parameters were slightly increased in the verum compared to the placebo group (IDCH, IDFC, L4AB, L4PN, L4TG) at baseline. After 2-way ANOVA analysis and Bonferroni correction, ABA1 (*p = 0.030*), IDPN (*p = 0.022*), IDCH (*p = 0.004*), IDFC (*p = 0.004*), IDAB (*p = 0.022*), and V4FC (*p = 0.035*) remained significantly changed upon intervention. Interestingly, a few changes were maintained in the wash-out phase of 6 weeks, including ABA1, IDAB, IDCH, IDFC, and IDPN. Therefore, intervention with aronia juice leads to several changes in human lipoprotein metabolism, and these changes are maintained even for several weeks after ending the intervention (Fig. 7A).

To reveal if tolerability for aronia juice can be detected using plasma metabolites or lipoproteins as biomarkers, we carried out additional analyses (Additional file 5). Regarding the tolerability groups, no differences in the metabolic profiles could be observed between Vt and Vc at baseline. Taking into account all time points, treatment, and tolerability in a 3-way ANOVA analysis with Bonferroni correction, we found formic acid (*p = 0.005*) to be significantly lower in Vt compared to Vc. A significant increase of formic acid was observed in Vt from baseline to after the intervention (*p = 0.034*). In addition, glutamine (*p = 0.009*) was significantly lower in Vc compared to Vt after the intervention. However, no significant changes in the metabolic profile were observed longitudinally for Vc.

Separated for tolerability, initial significant differences in lipoprotein profile between Vt and Vc and after the intervention were detected and related to several LDL-related parameters. In 3-way ANOVA and after Bonferroni correction, L2AB (*p = 0.043*), L2CH (*p = 0.008*), L2FC (*p = 0.009*), L2PL (*p = 0.013*), L2PN (*p = 0.043*), and L3FC (*p = 0.043*) remained significantly up in Vt (Additional file 4). Importantly, only in Vt, the longitudinal changes of lipoprotein profile upon intervention could be observed, with the same LDL-related parameters showing significant changes with time in 3-way ANOVA and after Bonferroni correction—L2AB (*p = 0.048*), L2CH (*p = 0.009*), L2FC (*p = 0.010*), L2PL (*p = 0.014*), L2PN (*p = 0.048*), and L3FC (*p = 0.038*) (Fig. 7B).

### Metabolomic outcome fecal samples

NMR-based metabolomic analyses of the fecal samples was performed to analyze metabolites and breakdown products from polyphenols to assess the intake, digestibility, and cleavage of polyphenols. Thereby, metabolic shifts in stool samples were observed for all groups over the three time points; however, none of these shifts remained significant after Bonferroni correction (Additional file 6).

Three-way ANOVA analysis revealed a significant increase of gallic acid (*p = 0.007*), a breakdown product of polyphenols, in the verum group; however, this significance did not remain after Bonferroni correction (*p = 0.300*).

## Discussion

### Summary of the results

In this clinical randomized single-blinded placebo-controlled parallel-design intervention trial, the effects of 6 weeks of aronia juice consumption followed by another 6 weeks of wash out on the gut microbial composition and associated metabolic outcome were investigated. Since striking differences in the tolerability of the aronia juice occurred, the verum group was divided into a tolerated and a complaints group for data analysis. Although significant changes in alpha diversity indices were only observed in Vt over time, the change in microbial composition led to a completely different predicted metabolic flux between Vt and Vc. In Vt, six of the 40 most likely metabolites were cholates, thus associated with the bile acid and lipoprotein metabolism. Metabolomics data on lipoprotein profiles showed alteration in several subfractions of LDL and VLDL molecules after the intervention in Vt that disappeared again in the wash out phase. In Vc, ammonium and carbon dioxide as well as nine O-glycans that are known to make up the mucus system were among the 40 most likely metabolites processed by gut microbes. This finding underpins changes due to gastrointestinal complaints in the Vc group.

### Acceptance and tolerability of aronia juice

*Aronia melanocarpa*, with its high polyphenol content, including considerable concentrations of tannins, is known for its astringent and bitter taste which is rather unappealing to certain people [30]. It has been discussed previously that the acceptance of aronia products may be limited to people who like its specific taste. However, compared to other polyphenol-rich beverages such as sea buckthorn, acerola, or cranberry, aronia showed the highest consumer acceptance [31]. To avoid unnecessary drop out due to individual taste preference, we only included females who reported liking bitter and astringent drinks. However, in our cohort, some participants experienced physical discomfort after prolonged juice consumption. To our knowledge, gastrointestinal discomfort has not been reported previously after natural aronia product consumption.

Tannins are known to interact with diverse food molecules such as proteins and polysaccharides, and with salivary proteins ending up in astringency and bitterness. Astringent mouthfeel is also discussed as a consequence of lubrication failure due to low saliva flow rate and xerostomia [65]. In our cohort, we could not identify internal factors contributing to elevated taste perception. However, tolerability could be slightly enhanced by advising the participants to dilute the juice with water or consuming it together with meals which ensured compliance despite reported complaints. The effect of food combination and meal planning strategies might be explained by a tannins-protein and tannins-polysaccharides interaction. Prior research noted individual variations in polyphenol bioavailability and catabolism [61, 66, 67] related to food composition, intestinal enzyme activity, transit time, and colonic microbiota composition [68–70], which might contribute to differences in metabolic effects of polyphenols.

### Aronia juice and gut microbiota modulation

In our study, the alpha diversity expressed as Shannon index and richness increased only in Vt over the study period, while no significant differences were observed for Vc, V in total, and P. Regarding beta diversity, we could not observe significant changes; however, in Vt, *Anaerostipes* and *Bacteroides* species seemed to be affected during the study period. Although no significant changes in microbial composition were observed, the MICOM model-based flux balance analysis of keystone taxa revealed pronounced differences in expected metabolites produced by the gut microbiota between Vt and Vc. In Vc, an increased gas production could be observed which underpins the complaints reported by this group. While a lot of CO_2_ was exported, a lack of CO_2_-consuming microbes was identified in the network. Moreover, high metabolic mucosal activity was shown which might be explained by the changed conditions caused by aronia juice polyphenols. The intestinal microbiome of Vc appeared to handle these substances poorly. In contrast, a stable network between the gut microbes of Vt and cholates associated with lipid metabolism was shown, and the import and export of fermentation products seem to be rather balanced. Apparently, this microbial community was more advantageous for the turnover of aronia juice polyphenols. A changed composition of the network between *Bacteroides* and *Anaerostipes* could be of importance for tolerability. In summary, a potential impact of the aronia juice polyphenols on the modulation of microbial composition can be assumed.

An increase in alpha diversity after treatment with *Aronia melanocarpa* polysaccharides has previously been observed in mice [71]. Also, an increase in *Anaerostipes* [72] and *Bacteroides* [73] after the application of aronia polyphenols has already been shown in in vitro models as well as in a clinical placebo-controlled intervention trial [61]. Istas et al. [61] found no significant changes in gut microbiota diversity in a human placebo-controlled intervention study with polyphenol-rich aronia extract and whole fruit aronia berry powder in healthy adult men. However, the consumption of aronia extract led to an increase in the abundance of *Anaerostipes* (+ 10.6%), while the intake of the whole fruit powder increased *Bacteroides* abundance significantly by 193%. Moreover, Wu et al. [72] reported aronia polyphenols to modulate microbial composition in an in vitro study. They investigated the mechanistic effects of aronia juice polyphenols on microbial composition in a simulator of the human intestinal microbial ecosystem (SHIME) and a Caco-2/endothelial cell coculture model and found an increased abundance of *Anaerostipes* after aronia juice application. Yu et al. [73] found black chokeberry anthocyanins to significantly increase the relative richness of *Bacteroides* and others in an in vitro model. These findings are in line with our observed trend in the increased abundance of these two genera *Anaerostipes* and *Bacteroides* in the Vt group.

Concerning single polyphenols contained in the polyphenol blend of *Aronia melanocarpa* several experimental studies in animal and cell models showed an impact of these polyphenols on the gut microbiome, thereby primarily proposing beneficial effects. Studies on the impact of procyanidins (e.g., [74, 75], anthocyanins (e.g., [76, 77]), and phenolic acids (e.g., [78, 79]) showed modification in the gut microbiota that was associated with reduced risk for atherosclerosis, gestational diabetes mellitus, inflammatory pathways, diet-induced obesity, and non-alcoholic fatty liver diseases, respectively. However, in our study, we could not confirm microbial or metabolic outcomes pointing to beneficial cardiovascular or antiatherosclerosis effects in healthy young females since LDL and VLDL subfractions increased.

In total, available literature supports the beneficial benefits of aronia juice as a food component or their major polyphenols on gut microbiota. On contrary, we observed intolerance reactions in half of our participants leading to alterations in metabolic outcomes. We assume that changes in gut microbial composition contribute to intolerability symptoms and impact gut health, as the microbiota is involved in the mucus production supporting the gut barrier function, and endothelial cell integrity [15].

### Aronia juice’s impact on gut health

We found a striking number of predicted O-glycans in the complaints group. O-glycans are main components of the intestinal mucins that form the mucus layer of the gut epithelium. Thereby, core 3-derived O-glycans are primarily expressed in the colon. O-Glycans play a central role in maintaining intestinal health by influencing barrier integrity, preventing pathogen adhesion, modulating the immune response, and contributing to eubiosis by shaping the mucin environment [80, 81]. In this regard, *Bacteroides* species *Lachnoclostridium* [22] and *Akkermansia* [72] are associated with mucus modulation properties and are stimulated after aronia polyphenol treatment. Taira et al. [82] found increased fecal mucin, improved intestinal barrier function and reduction of dysbiosis after aronia consumption in rats. Most interestingly, tannic acid, a prominent aronia polyphenol, shows a strong affinity to mucin production [83], suggesting a potential impact in our observation. Moreover, recent research demonstrated that aronia berry powder improves intestinal barrier function in a cell model of chronic colonic inflammation by modulation of tight junction expression, but isolated polyphenols, as surrogates of the microbiota-derived catabolites of aronia berry, did not replicate this effect [84].

While previous literature emphasizes the positive effects of polyphenols on mucus production, our study associates the predicted elevation of O-glycans with gastrointestinal disturbances*.* We assume a shift in the microbial profile induced by polyphenol ingestion and an accompanying adaption in immune response [32]. The increased production of O-glycans through microbiome modulation in Vc may serve to protect the epithelium from irritation caused by polyphenol overconsumption. Microbiota might respond to elevated polyphenol exposure with upregulation of O-glycan production to potentially mitigate any adverse effects on the gut epithelium.

### Alterations in plasma metabolite profile

In our cohort, plasma glutamine was significantly higher in Vt post-intervention, aligning with MICOM model-based flux balance analysis predictions indicating L-glutamine as one of the metabolites derived from gut microbiota. Glutamic acid, a precursor of L-proline and L-arginine, is obtained from food and endogenously synthesized from glutamine, α-ketoglutarate, and pyroglutamic acid in human metabolism [85]. The model-based flux balance analysis of keystone taxa predicted three metabolites (L-glutamine, 2-oxoglutarate, L-proline) associated with glutamic acid metabolism, linking the higher glutamine levels in Vt to metabolic processes triggered by changes in the gut microbiota.

Moreover, NMR metabolomics analysis revealed significantly higher formic acid concentration in Vc after the intervention. Formic acid plays an important role in human metabolism and serves as a metabolic mediator between the organism, diet, and gut microbiota. It is produced endogenously from the cleavage of serine and glycine, the catabolism of tryptophan and choline, and the synthesis of sterol and cholesterol. However, about 50% of the circulating formic acid pool is attributed to anaerobic fermentation by gut microbes. Formic acid is oxidized in human cells to carbon dioxide (CO_2_) [86]. This CO_2_ may have caused bloating and intestinal discomfort reported by the participants of Vc. The observed higher formic acid levels in Vc in plasma support higher CO_2_ production proposed by the prediction model (Fig. 5).

The intervention with aronia juice led to several changes in human lipoprotein metabolism, and some of these changes were maintained even for several weeks after ending the intervention. Several proatherogenic subclasses of LDL and VLDL increased in Vt after aronia juice consumption and decreased again during the washout phase which was also associated with several predicted cholates as microbial metabolites and increased fecal glycerol excretion. This suggests an effect of polyphenols on cholesterol metabolism via bile acid biosynthesis [87] as primary bile acids are modified by gut microbes, resulting in the formation of secondary bile acids which is accompanied by changes in the bioavailability and bioactivity of bile acids [88–90]. Secondary bile acids intricately influence lipoprotein metabolism through diverse pathways. They participate in the enterohepatic circuit, act as ligands for the nuclear farnesoid X receptor (FXR), and regulate cholesterol metabolism and gene expression related to cholesterol synthesis and transport [91]. Additionally, secondary bile acids serve as signaling molecules, activating FXR and Takeda G protein-coupled receptor 5 (TGR5) to jointly regulate glucose and lipid metabolism, energy expenditure, and inflammation. Experimental studies link the gut microbiome and bile acids to cardiovascular disease risk factors, including atherosclerosis [92]. However, precise mechanisms require further exploration. In addition, glycerol in stool might point to a higher cleavage of triglycerides and endogenous triglyceride turnover [93]. Of note, an increase in cholates has also been reported after high polyphenol consumption within the Mediterranean dietary patterns [94].

Changes in plasma lipoprotein levels after polyphenol consumption have been observed in healthy subjects before [95]. In contrast to our results, Rahmani et al. [96] found a significant increase in HDL after aronia consumption and decrease in cholesterol and LDL in a meta-analysis of seven studies. Interestingly, they found a significant reduction in triglyceride levels when aronia was administered in higher doses (300 mg/day), whereas others used lower doses of 100 ml/day [97]. We administered 200 ml of aronia juice a day, which is in line with other interventions [33–35]. Thus, possibly hermetic effects may have contributed to these diverse outcomes, whereby the polyphenolic composition of the aronia products may differ by the growing and processing conditions of the plants [27, 28]. Importantly, sex differences in lipoprotein metabolism have been described previously. Petrovic et al. [97] reported elevated triglyceride levels in women, but not in men. The significant increase in VLDL-particles and a consecutive change to baseline level in the washout period could therefore be a gender-specific phenomenon. To our knowledge, there are currently no studies available reporting on VLDL-levels in the context of aronia or polyphenol consumption.

In mouse models, aronia powder has demonstrated a positive effect on lipid metabolism by attenuating de novo lipogenesis in hepatocytes of mice with nonalcoholic fatty liver disease [98]. Despite potential induction of hepatic de novo lipogenesis by high fructose ingestion and microbial fermentation of fructose to acetate in the gut [99], our study suggests to exclude the influence of fructose on lipoprotein alteration in our cohort. The observed increase in LDL and VLDL were only observed in Vt, with no changes in Vc or P, despite comparable additional fructose consumption. An elevation of VLDL has also been reported as a result of the mobilization of fatty acids in adipose tissue [100]. This might underpin polyphenols’ impact on adipose tissue physiology and their lipid oxidation properties within the adipocyte [101, 102].

### Strengths and limitations

Since we divided the study cohort into tolerability groups, the study population is small. A larger sample size would strengthen the revealed associations. Our comparative power analyses of microbial taxa and predicted metabolic fluxes suggest that a bigger cohort and more samples would have been necessary to determine significance, but our results were rather robust against the selected sub-groupings within our dataset. Furthermore, the investigation was conducted in humans under their habitual lifestyle conditions. Even though we aimed to document all possible influencing factors, also other stressors the participants might have encountered during the study period may have influenced the outcome. We further need to acknowledge that possibly other unknown facts before conducting the study may have led to differences in the tolerability. However, the strength of this study is that we included females only. In light of sex differences in lipid metabolism, the homogenous group of females is an advantage in data interpretation. Including only women enhances gender-specific insights but may limit the generalizability of findings to the broader population.

## Conclusions

The interindividual differences in the tolerability of aronia juice were associated with variations in gut microbiota derived metabolites and the metabolic profile in plasma and stool. Intolerance symptoms were reflected in the microbiota-derived metabolites indicating the adaption of gut microbes to the aronia polyphenols. In contrast to previous findings, an increase in lipoprotein profile due to modification of the gut microbiome through aronia juice consumption was detected. These findings suggest that it may be important to explore personalized recommendations for the level of polyphenol consumption to benefit from their potential health benefits. Further research is needed to identify possible microbial contributors to polyphenol pathways in the human gut. Dose-dependent effects of aronia polyphenols should be tested in accordance with individual factors such as age, sex, and defined biomarkers. Even though we could reproduce some of the findings on aronia polyphenols on the gut microbiome diversity and specific genera in a human female cohort, we could not confirm favorable effects on lipoprotein profiles in this healthy cohort. Further studies should investigate larger cohorts including males and consider testing potential hermetic doses to further define the interindividual tolerability differences.

### Supplementary Information

**Additional file 1.** CONSORT 2010 Flow Diagram. Enrollment progress of study participants for each group including number of study participants and losses of participants together with reasons.

**Additional file 2.** Power Calculation. Calculation of effect sizes and power of microbiome data and predicted flux of metabolites.

**Additional file 3.** Metadata. Metadata information on the study population including group allocation, age, BMI, smoking and nicotine dependency at baseline, and weight, waist circumference and detailed dietary information (e.g., diversity, energy, protein, fat, carbohydrates) of all three measurement points.

**Additional file 4.** Lipoproteins. ANOVA lipoproteins: Changes in lipoprotein profiles as detected by NMR-metabolomics analysis, Results of 2-way and 3-way ANOVA and significant*p*-values.

**Additional file 5.** Plasma metabolites. ANOVA plasma metabolites: Changes in plasma metabolite profiles as detected by NMR-metabolomics analysis, Results of 2-way and 3-way ANOVA and significant*p*-values.

**Additional file 6.** Stool metabolites. ANOVA stool metabolites: Changes in stool metabolite profiles as detected by NMR-metabolomics analysis of fecal samples, Results of 2-way and 3-way ANOVA and significant *p*-values.

## Data Availability

All data generated or analyzed during this study are included in this published article and its additional files and can be accessed via the databases listed below. Raw sequencing data obtained from amplicon-based sequencing (technical sequences including adaptor sequences, linker sequences, and barcode sequences were removed) used in this paper can be found in the European Nucleotide Archive (ENA): PRJEB64786 (https://www.ebi.ac.uk/ena/browser/home). The datasets on microbiome generated and analyzed during the current study are available in GitHub repository, https://github.com/Christine-Moissl-Eichinger/aronia/. The datasets on stool and plasma metabolomics generated and analyzed during the current study are available at MetaboLights under the accession number MTBLS7661 (https://www.ebi.ac.uk/metabolights/). *Comment: Until the data release on MetaboLights, the data is provided via nexcloud and can be assessed at the following links: Stool data: *https://box.medunigraz.at/s/iTZo2GmMS6Z7YsD*, plasma data: *https://box.medunigraz.at/s/9mBAT26d8YLDNRz

## Abbreviations

ABA1: Apo-B100/Apo-A1 ratio
H3A2: HDL-3 Apo-A2
IDAB: IDL Apo-B100
IDCH: IDL cholesterol
IDFC: IDL free cholesterol
IDPL: IDL phospholipids
IDPN: IDL particle number
L4AB: LDL-4 Apo-B100
L4CH: LDL-4 cholesterol
L4FC: LDL-4 free cholesterol
L4PL: LDL-4 phospholipids
L4PN: LDL-4-particle number
L4TG: LDL-4 triglycerides
L5AB: LDL-5 Apo-B100
L5CH: LDL-5-cholesterol
L5FC: LDL-5 free cholesterol
L5PL: LDL-5 phospholipids
L5PN: LDL-5 particle number
LDHD: LDL-cholesterol/ HDL-cholesterol ratio
MICOM: Microbial community (flux balance analysis)
P: Placebo
RSV: Ribosomal sequence variant
TBPN: Total particle number
TPAB: Total Apo-B100
V: Verum
V3CH: VLDL-3 cholesterol
V3FC: VLDL-3 free cholesterol
V3PL: VLDL-3 phospholipids
V3TG: VLDL-3 triglycerides
V4CH: VLDL-4 cholesterol
V4FC: VLDL-4 free cholesterol
V4PL: VLDL-4 phospholipids
V4TG: VLDL-4 triglycerides
Vc: Verum complaints
VLAB: VLDL Apo-B100
VLCH: VLDL cholesterol
VLPN: VLDL particle number
Vt: Verum tolerated
