# Supplementary figures and images for: Interindividual differences in aronia juice tolerability linked to gut microbiome and metabolome changes—secondary analysis of a randomized placebo-controlled parallel intervention trial
Source: Microbiome. 2024 Mar 9;12:49. doi: 10.1186/s40168-024-01774-4 (PMC10924357; doi:10.1186/s40168-024-01774-4)

## CONSORT 2010 Flow Diagram

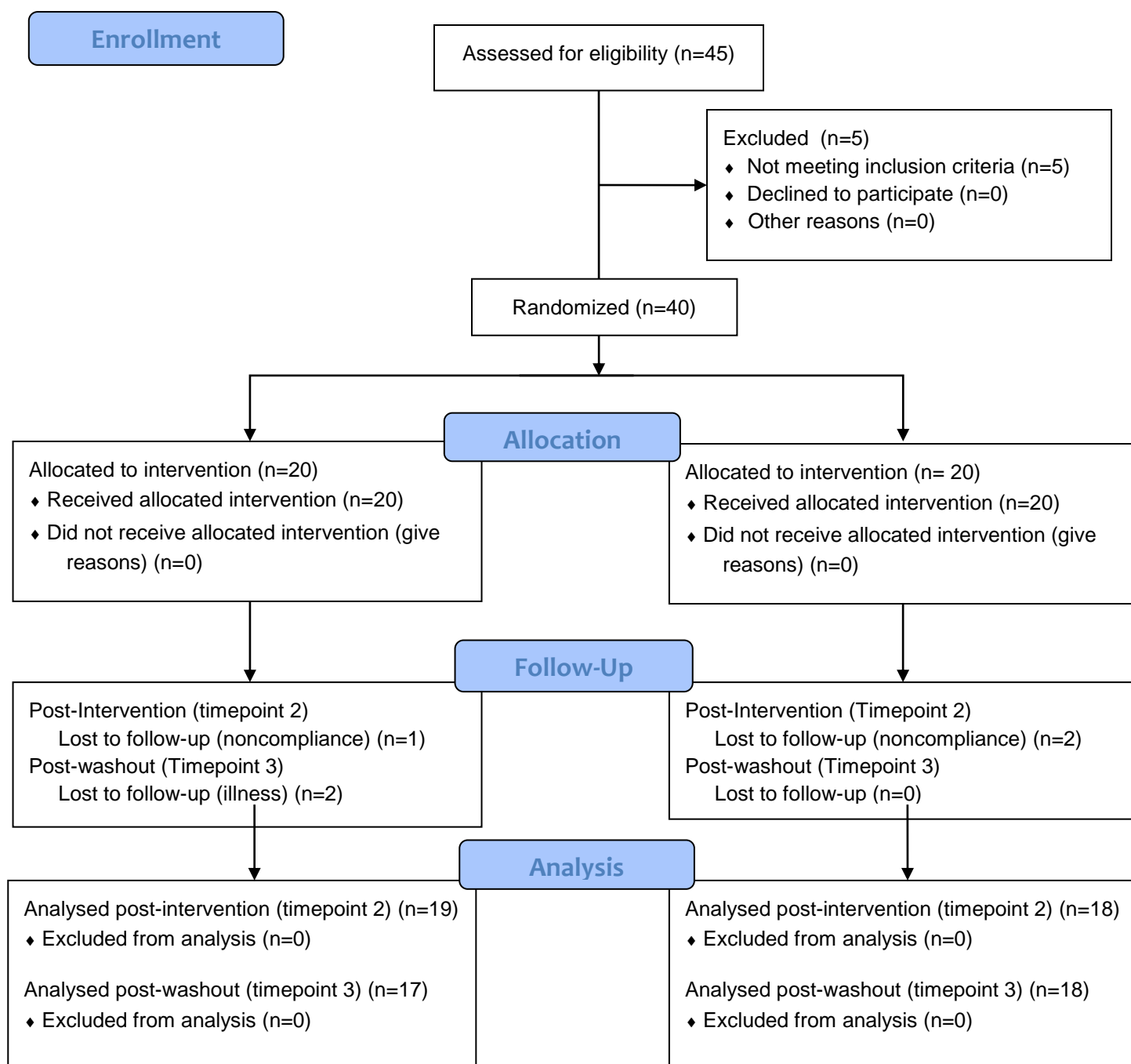

Supplement: Supplementary file 2 — Additional file 1. CONSORT 2010 Flow Diagram. Enrollment progress of study participants for each group including number of study participants and losses of participants together with reasons. [file 40168_2024_1774_MOESM1_ESM.pdf]
