# Supplementary material for: Interindividual differences in aronia juice tolerability linked to gut microbiome and metabolome changes—secondary analysis of a randomized placebo-controlled parallel intervention trial
Source: Microbiome. 2024 Mar 9;12:49. doi: 10.1186/s40168-024-01774-4 (PMC10924357; doi:10.1186/s40168-024-01774-4)
Supplement: Supplementary file 3 — Additional file 2. Power Calculation. Calculation of effect sizes and power of microbiome data and predicted flux of metabolites. [file 40168_2024_1774_MOESM2_ESM.pdf]

We conducted effect size and power calculations with q2-evident (https://github.com/biocore/evident) on the microbiome data and the predicted flux of metabolites in our study. As suggested, the main groups “Placebo” (n=56) and “Verum” (n=56) were compared to the finer and smaller groupings of “Pc t1 (n=1), Pc t2 (n=1), Pc t3 (n=1), Pt t1 (n=17), Pt t2 (n=17), Pt t3 (n=17), Vc t1 (n=10), Vc t2 (n=10), Vc t3 (n=9), Vt t1 (n=9), Vt t2 (n=9), Vt t3 (n=8) we used in our datasets (Pc = Placebo complaints, Pt = Placebo tolerated, Vc = Verum complaints, Vt = Verum tolerated).

First, we conducted univariate power analysis based on microbial diversity (Shannon entropy) for the main group “Placebo” and “Verum”, and visualized the results as power curves for a target power (1 – beta) of 0.8, at an alpha of 0.01, 0.05, and 0.1 for 10 to 1000 observations in 100 steps.

In this case, the intersection of the power curve with the target power (1 – beta) was around ~100 observations for an alpha at 0.05 (see Fig.1).

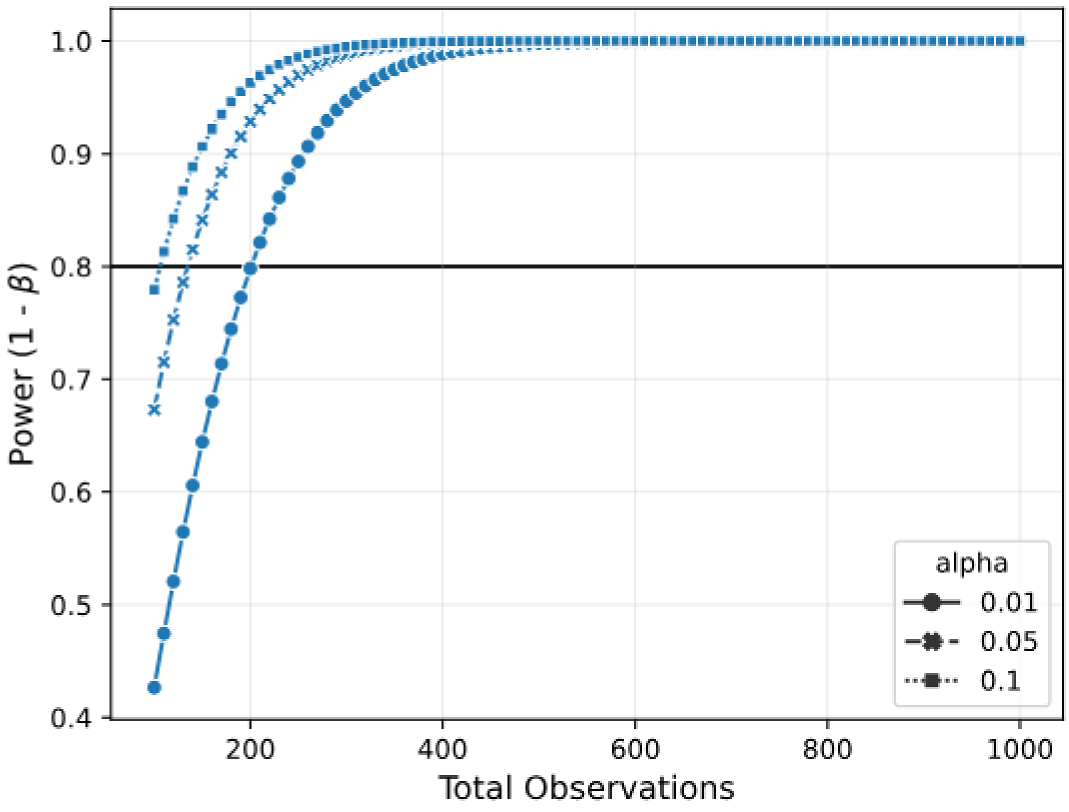

Fig. 1: Power curve based on univariate power calculations for microbial diversity using Shannon entropy for the groups Placebo and Verum at different alpha’s.

We did the same for the finer and smaller groupings we used in our study. According to this plotted power curve, this analysis revealed a shift of the power curve with the target power (1 – beta) to ~180 observations for an alpha at 0.05 (see Fig. 2).

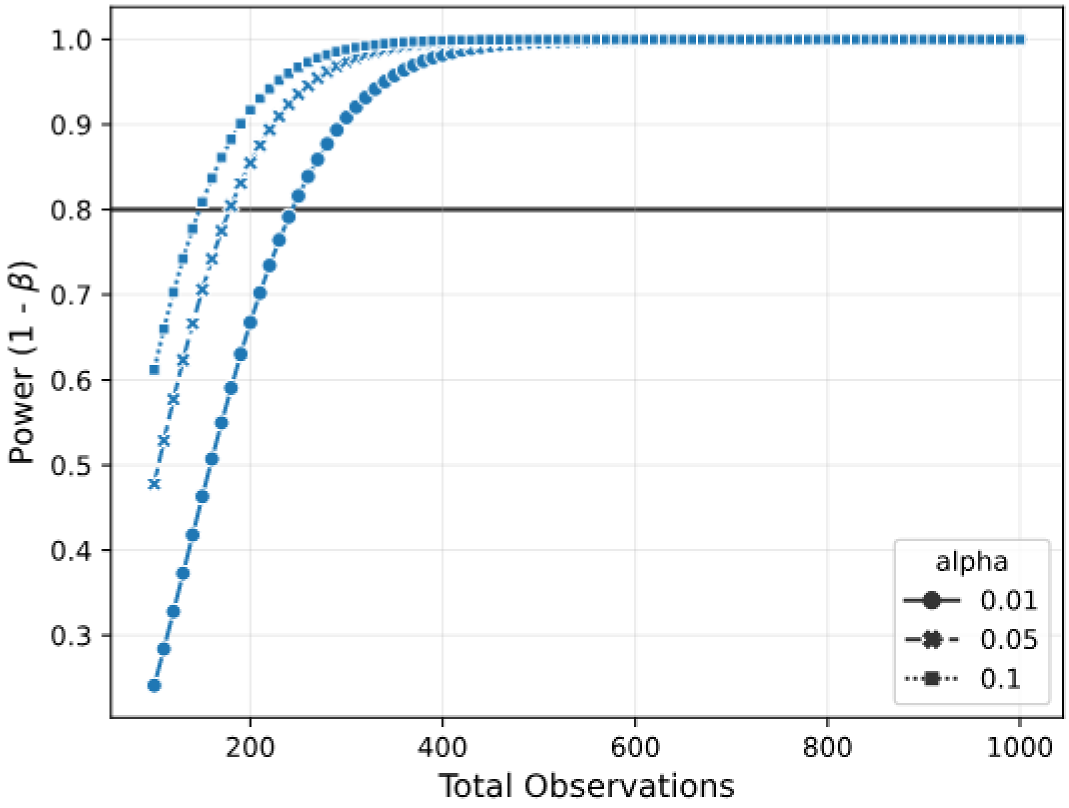

Fig. 2: Power curve based on univariate power calculations for microbial diversity using Shannon entropy for the groups Placebo complaints, Placebo tolerated, Verum complaints and Verum tolerated at all three timepoints and at different alpha's.

In addition to the univariate power analysis we also conducted a multivariate power analysis based on distances in microbial composition (Bray-Curtis distances) for the different groups in our datasets.

For the main group “Placebo” and “Verum” the multivariate power analysis was visualized as power curves for a target power (1 – beta) of 0.8, at an alpha of 0.01, 0.05, and 0.1 for 10 to 1000 observations in 100 steps.

Regarding microbial distances, the intersection of the power curve with the target power (1 – beta) dropped to ~100 observations for an alpha at 0.05 (see Fig.3) in comparison to the univariate analysis for microbial diversity we presented above.

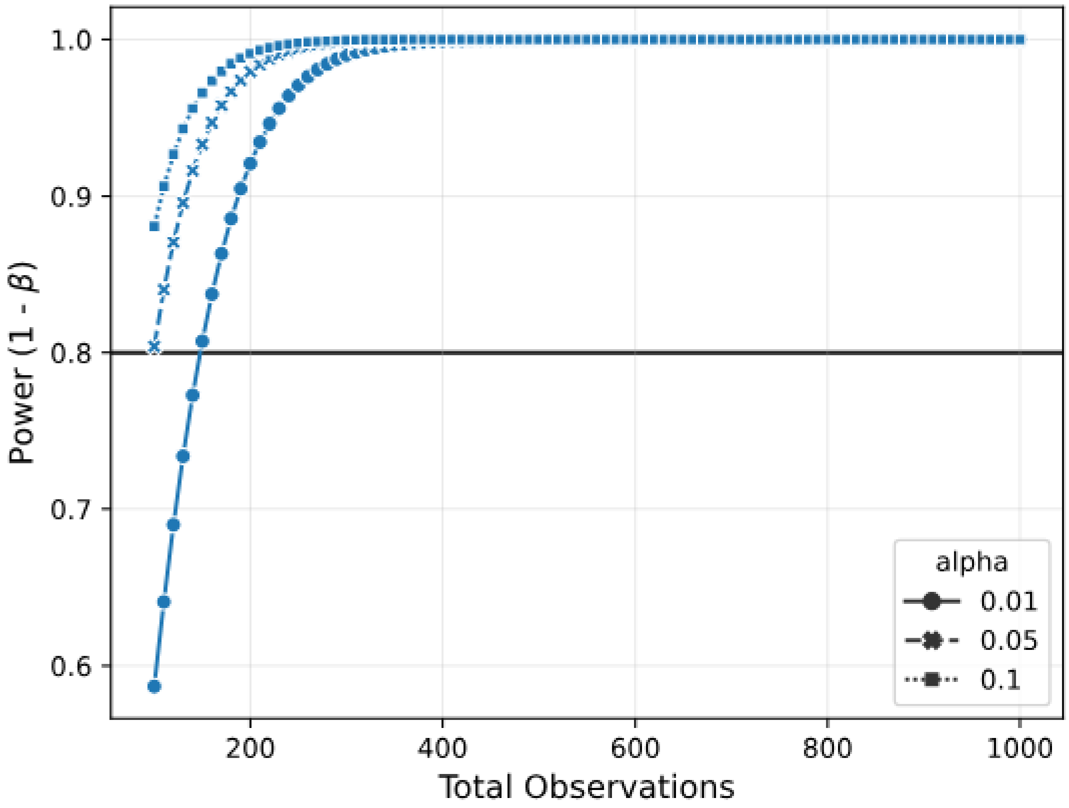

Fig. 3: Power curve based on multivariate power calculations for microbial composition using Bray-Curtis distances for the groups Placebo and Verum at different alpha’s.

Again, we did the same analyses for the finer and smaller groupings we used in our study. According to this plotted power curve, this analysis revealed only a slight shift of the power curve with the target power (1 – beta) to >100 observations for an alpha at 0.05 (see Fig. 4).

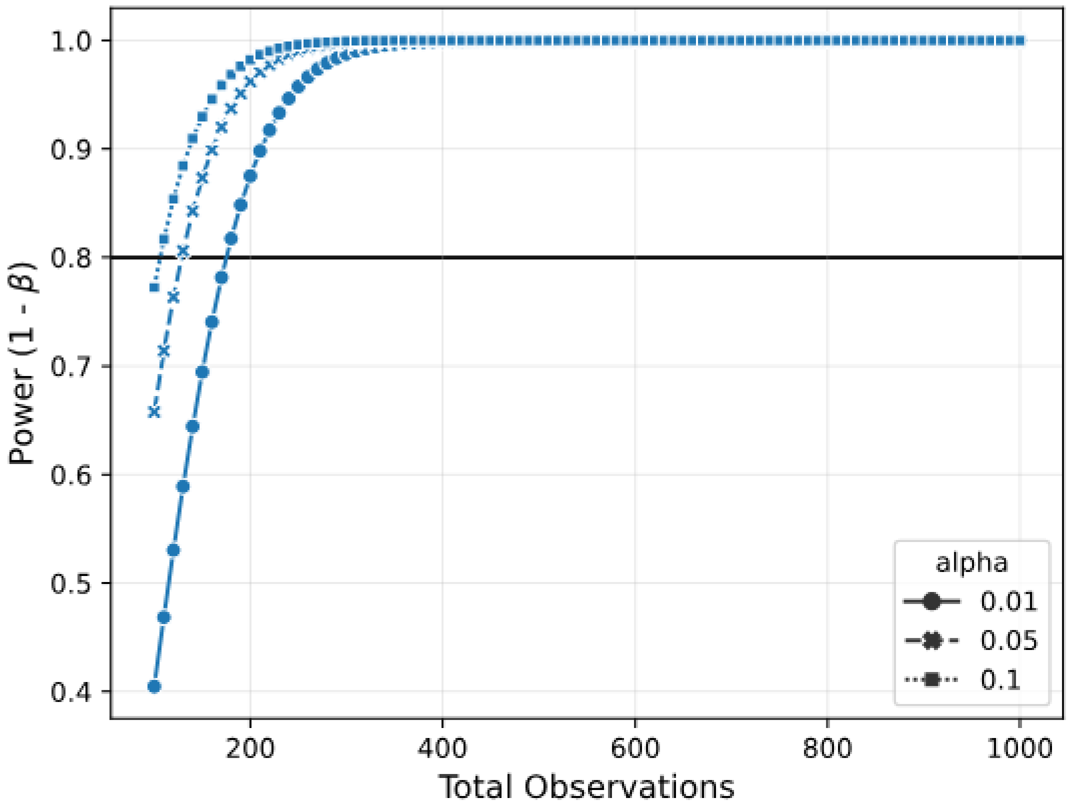

Fig. 4: Power curve based on multivariate power calculations for microbial composition using Bray-Curtis distances for the groups Placebo complaints, Placebo tolerated, Verum complaints and Verum tolerated at all three timepoints and at different alpha’s.

As a conclusion for the taxa specific microbiome analysis, we also calculated all pairwise effect sizes for each of the used metadata categories. The following table shows the results for Shannon entropy (Table 1) and Bray-Curtis distances (Table 2) we used.

This analysis revealed that the effect size (based on Cohen’s d) was not the highest for the main groups and higher effect sizes could be achieved even at the finer and smaller groupings we used in the study.

Table 1: Univariate effect size calculations by the used metadata categories based on microbial diversity expressed by Shannon entropy.

effect_size	metric	column	group_1	group_2
0.979301075	cohens_d	group2	Verum tolerated t2	Verum tolerated t3
0.947064767	cohens_d	group2	Placebo tolerated t2	Verum tolerated t2
0.871167878	cohens_d	group2	Placebo tolerated t2	Verum tolerated t1
0.818170816	cohens_d	group2	Verum tolerated t1	Verum tolerated t3
0.748848172	cohens_d	group2	Placebo tolerated t3	Verum tolerated t2
0.711200236	cohens_d	group2	Placebo tolerated t1	Verum tolerated t2
0.708582034	cohens_d	group2	Placebo tolerated t3	Verum tolerated t1
0.695770274	cohens_d	group2	Placebo tolerated t2	Verum complaints t1
0.660562828	cohens_d	group2	Placebo tolerated t1	Verum tolerated t1
0.602628717	cohens_d	group2	Placebo tolerated t3	Verum complaints t1
0.585562733	cohens_d	group2	Verum complaints t2	Verum tolerated t2
0.567999367	cohens_d	group2	Verum complaints t1	Verum tolerated t3
0.539707058	cohens_d	group2	Verum complaints t3	Verum tolerated t2
0.537963365	cohens_d	group2	Placebo tolerated t1	Verum complaints t1
0.530228348	cohens_d	group2	Verum complaints t2	Verum tolerated t1
0.48647091	cohens_d	group	Placebo	Verum
0.473096718	cohens_d	group2	Verum complaints t3	Verum tolerated t1
0.413877747	cohens_d	group2	Verum complaints t1	Verum complaints t2
0.400965471	cohens_d	group2	Placebo tolerated t2	Verum complaints t3
0.350504895	cohens_d	group2	Verum complaints t1	Verum complaints t3
0.313212502	cohens_d	group2	Placebo tolerated t3	Verum complaints t3
0.306124158	cohens_d	group2	Verum complaints t3	Verum tolerated t3
0.275630496	cohens_d	group2	Placebo tolerated t2	Verum complaints t2
0.214231665	cohens_d	group2	Placebo tolerated t3	Verum complaints t2
0.209918319	cohens_d	group2	Placebo tolerated t1	Verum complaints t3
0.175545641	cohens_d	group2	Placebo tolerated t1	Placebo tolerated t2
0.168335633	cohens_d	group2	Verum complaints t2	Verum tolerated t3
0.130486958	cohens_d	group2	Placebo tolerated t1	Placebo tolerated t3
0.126303077	cohens_d	group2	Placebo tolerated t2	Verum tolerated t3
0.10115726	cohens_d	group2	Placebo tolerated t1	Verum complaints t2
0.098532579	cohens_d	group2	Verum complaints t2	Verum complaints t3
0.079389671	cohens_d	group2	Placebo tolerated t3	Verum tolerated t3
0.061757841	cohens_d	group2	Placebo tolerated t1	Verum tolerated t3
0.024641472	cohens_d	group2	Placebo tolerated t2	Placebo tolerated t3
0.011522069	cohens_d	group2	Verum complaints t1	Verum tolerated t2
0.006814042	cohens_d	group2	Verum complaints t1	Verum tolerated t1
0.006303862	cohens_d	group2	Verum tolerated t1	Verum tolerated t2

Table 2: Multivariate effect size calculations by the used metadata categories based on microbial composition expressed by Bray-Curtis distances.

effect_size	metric	column	group_1	group_2
1.056199715	cohens_d	group2	Placebo tolerated t1	Verum tolerated t1
1.00436733	cohens_d	group2	Placebo tolerated t1	Verum tolerated t2
0.939770994	cohens_d	group2	Placebo tolerated t2	Verum tolerated t1
0.894804396	cohens_d	group2	Placebo tolerated t2	Verum tolerated t2
0.826313524	cohens_d	group2	Placebo tolerated t3	Verum tolerated t1
0.815386649	cohens_d	group2	Placebo tolerated t1	Verum complaints t1
0.777426284	cohens_d	group2	Placebo tolerated t1	Verum complaints t3
0.77358479	cohens_d	group2	Placebo tolerated t3	Verum tolerated t2
0.725897823	cohens_d	group2	Placebo tolerated t2	Verum complaints t1
0.716389624	cohens_d	group2	Verum complaints t2	Verum tolerated t1
0.678377731	cohens_d	group2	Placebo tolerated t2	Verum complaints t3
0.653372832	cohens_d	group2	Verum complaints t2	Verum tolerated t2
0.627963423	cohens_d	group2	Verum tolerated t1	Verum tolerated t3
0.572492104	cohens_d	group2	Placebo tolerated t3	Verum complaints t1
0.568730168	cohens_d	group	Placebo	Verum
0.562394755	cohens_d	group2	Verum tolerated t2	Verum tolerated t3
0.517849199	cohens_d	group2	Placebo tolerated t3	Verum complaints t3
0.472197644	cohens_d	group2	Placebo tolerated t1	Verum tolerated t3
0.452225974	cohens_d	group2	Verum complaints t1	Verum complaints t2
0.429488292	cohens_d	group2	Verum complaints t2	Verum complaints t3
0.401840512	cohens_d	group2	Placebo tolerated t2	Verum tolerated t3
0.387891749	cohens_d	group2	Placebo tolerated t1	Verum complaints t2
0.363016478	cohens_d	group2	Verum complaints t1	Verum tolerated t3
0.361058653	cohens_d	group2	Verum complaints t3	Verum tolerated t1
0.341424624	cohens_d	group2	Verum complaints t3	Verum tolerated t3
0.32720916	cohens_d	group2	Placebo tolerated t2	Verum complaints t2
0.303442447	cohens_d	group2	Verum complaints t3	Verum tolerated t2
0.295571309	cohens_d	group2	Placebo tolerated t1	Placebo tolerated t3
0.247688746	cohens_d	group2	Placebo tolerated t2	Placebo tolerated t3
0.208236826	cohens_d	group2	Verum complaints t1	Verum tolerated t1
0.198380328	cohens_d	group2	Placebo tolerated t3	Verum tolerated t3
0.167967494	cohens_d	group2	Verum complaints t1	Verum tolerated t2
0.105504737	cohens_d	group2	Placebo tolerated t3	Verum complaints t2
0.098477219	cohens_d	group2	Verum complaints t1	Verum complaints t3
0.093216458	cohens_d	group2	Verum complaints t2	Verum tolerated t3
0.036429779	cohens_d	group2	Verum tolerated t1	Verum tolerated t2
0.029623976	cohens_d	group2	Placebo tolerated t1	Placebo tolerated t2

To address the potential constraints of our metabolic modelling we conducted the same analyses again, but in this case all individual analyses were based on the predicted metabolites for the different taxa from micom in our study.

Indeed, the power curves were shifted and in general more samples would be necessary to achieve similar power as for the standard microbiome analyses for univariate and multivariate measures. However, shifts between main and finer smaller groups for univariate (>300 observations) and multivariate (>100 observations) power analysis revealed only minor changes (see Fig. 5 – 8).

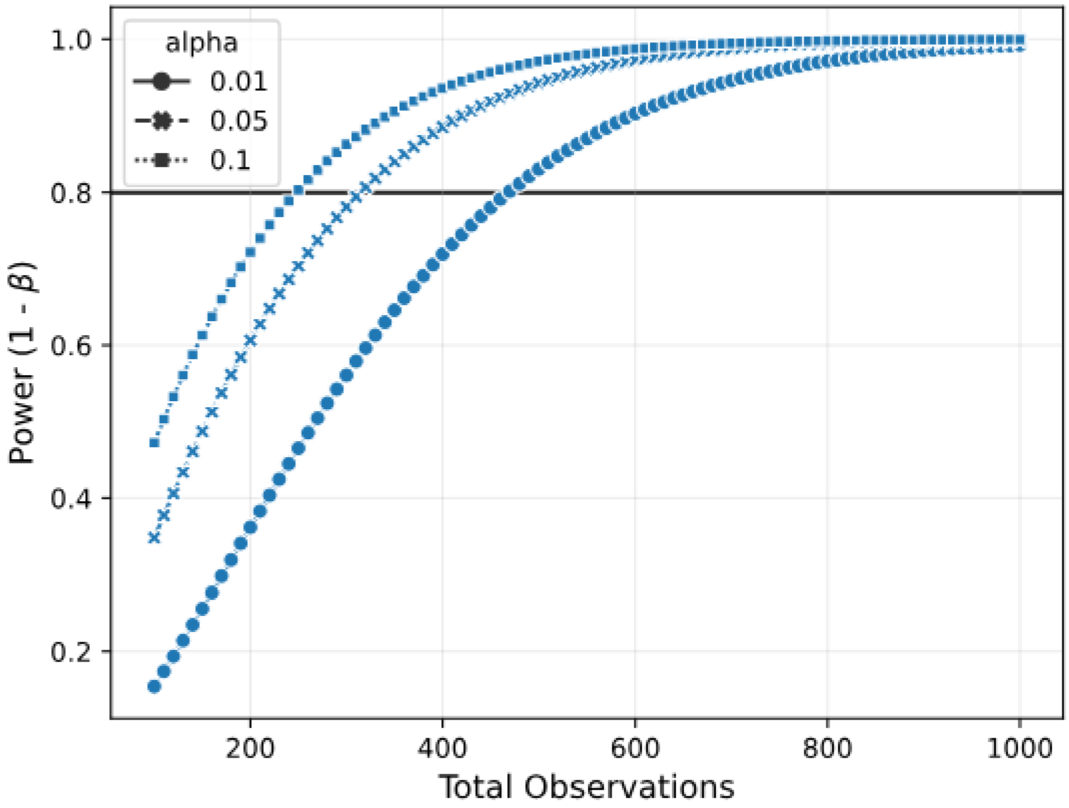

Fig. 5: Power curve based on univariate power calculations for predicted diversity of metabolites using Shannon entropy for the groups Placebo and Verum at different alpha's.

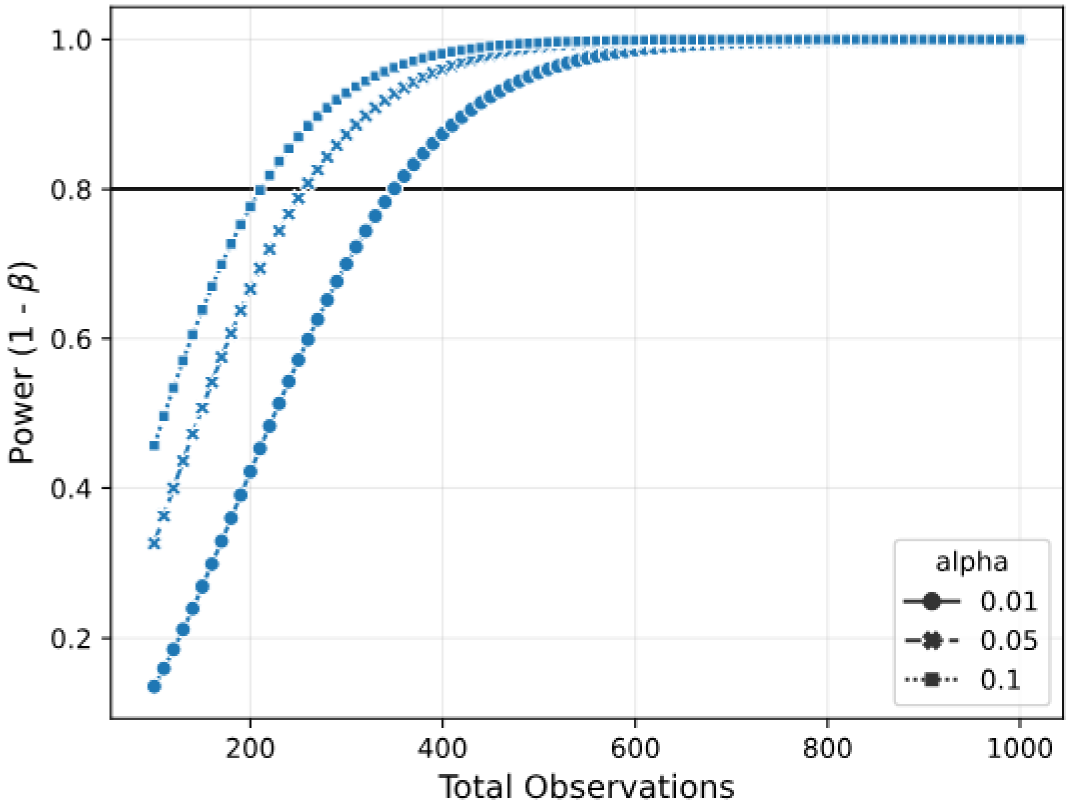

Fig. 6: Power curve based on univariate power calculations for predicted diversity of metabolites using Shannon entropy for the groups Placebo complaints, Placebo tolerated, Verum complaints and Verum tolerated at all three timepoints and at different alpha's.

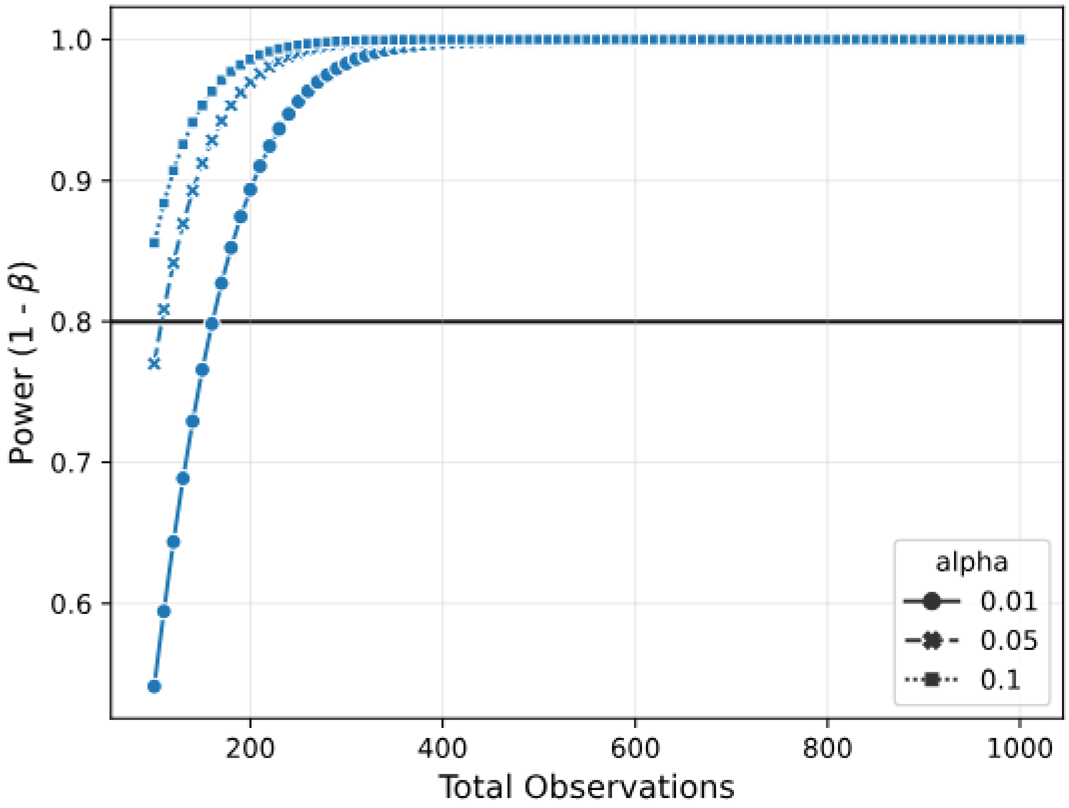

Fig. 7: Power curve based on multivariate power calculations for the composition of predicted metabolites using Bray-Curtis distances for the groups Placebo and Verum at different alpha’s.

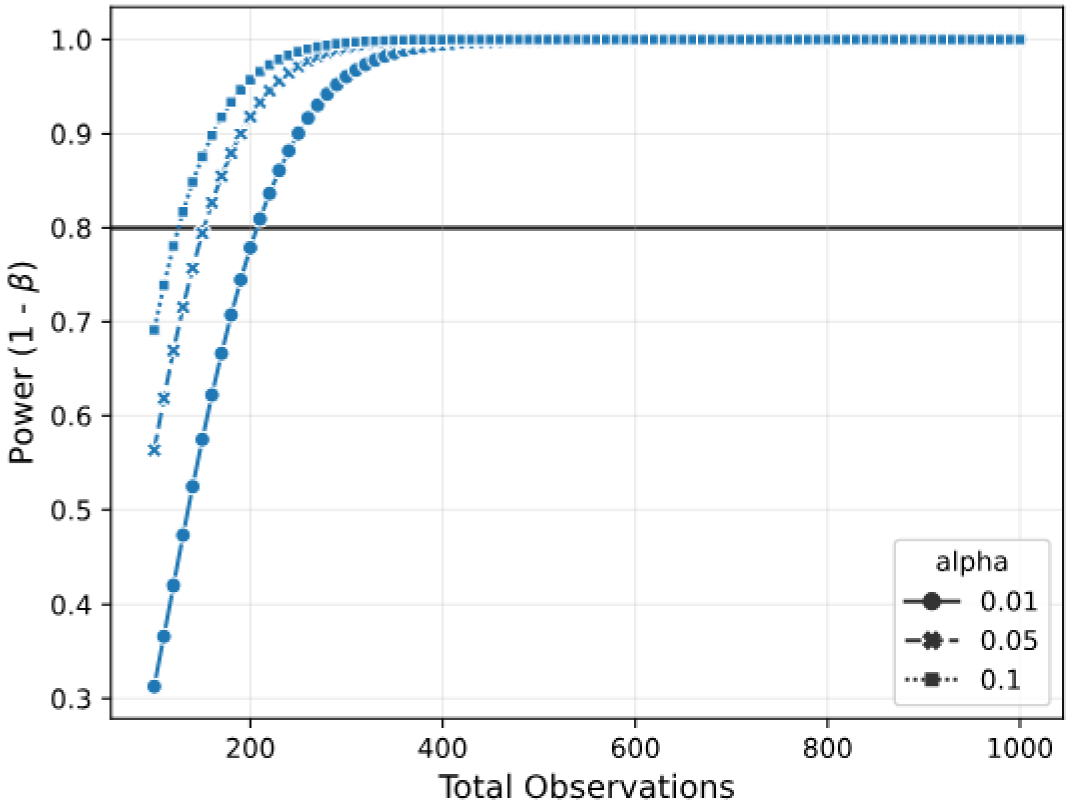

Fig. 8: Power curve based on multivariate power calculations for the composition of predicted metabolites using Bray-Curtis distances for the groups Placebo complaints, Placebo tolerated, Verum complaints and Verum tolerated at all three timepoints and at different alpha's.

As a conclusion for the predicted metabolic flux analysis, we also calculated all pairwise effect sizes for each of the used metadata categories again. The following table shows the results for Shannon entropy (Table 3) and Bray-Curtis distances (Table 4) we used.

Similarly, as seen before, this analysis revealed that the effect size (based on Cohen’s d) was not the highest for the main groups and higher effect sizes could be achieved even at the finer and smaller groupings we used in the study.

Table 3: Univariate effect size calculations by the used metadata categories based on predicted metabolite diversity expressed by Shannon entropy.

effect_size	metric	column	group_1	group_2
0.855399846	cohens_d	group2	Placebo tolerated t3	Verum tolerated t1
0.808006725	cohens_d	group2	Placebo tolerated t3	Verum complaints t1
0.646323406	cohens_d	group2	Verum complaints t3	Verum tolerated t1
0.645752722	cohens_d	group2	Placebo tolerated t3	Verum complaints t2
0.639823369	cohens_d	group2	Placebo tolerated t2	Verum tolerated t1
0.60573907	cohens_d	group2	Placebo tolerated t2	Verum complaints t1
0.565225941	cohens_d	group2	Verum complaints t1	Verum complaints t3
0.558536466	cohens_d	group2	Placebo tolerated t3	Verum tolerated t3
0.520462944	cohens_d	group2	Placebo tolerated t3	Verum tolerated t2
0.420223155	cohens_d	group2	Placebo tolerated t1	Placebo tolerated t3
0.419533024	cohens_d	group2	Placebo tolerated t2	Verum complaints t2
0.393170703	cohens_d	group2	Verum complaints t2	Verum complaints t3
0.337998505	cohens_d	group2	Placebo tolerated t1	Verum complaints t1
0.324387465	cohens_d	group2	Placebo tolerated t2	Verum tolerated t3
0.322048727	cohens_d	group2	Placebo tolerated t2	Placebo tolerated t3
0.318226829	cohens_d	group2	Placebo tolerated t3	Verum complaints t3
0.316925101	cohens_d	group	Placebo	Verum
0.309907476	cohens_d	group2	Placebo tolerated t1	Verum tolerated t1
0.303034702	cohens_d	group2	Verum complaints t3	Verum tolerated t3
0.295550827	cohens_d	group2	Placebo tolerated t2	Verum tolerated t2
0.269113482	cohens_d	group2	Verum complaints t3	Verum tolerated t2
0.228606008	cohens_d	group2	Verum complaints t1	Verum tolerated t3
0.212602067	cohens_d	group2	Verum complaints t1	Verum tolerated t2
0.203232416	cohens_d	group2	Verum tolerated t1	Verum tolerated t3
0.189050937	cohens_d	group2	Placebo tolerated t1	Verum complaints t2
0.181395847	cohens_d	group2	Verum tolerated t1	Verum tolerated t2
0.171299834	cohens_d	group2	Placebo tolerated t1	Placebo tolerated t2
0.159104375	cohens_d	group2	Placebo tolerated t1	Verum complaints t3
0.151410438	cohens_d	group2	Verum complaints t1	Verum complaints t2
0.108045924	cohens_d	group2	Placebo tolerated t1	Verum tolerated t3
0.107114163	cohens_d	group2	Verum complaints t2	Verum tolerated t1
0.102797423	cohens_d	group2	Placebo tolerated t1	Verum tolerated t2
0.080367773	cohens_d	group2	Verum complaints t2	Verum tolerated t3
0.074589319	cohens_d	group2	Verum complaints t2	Verum tolerated t2
0.06993847	cohens_d	group2	Verum complaints t1	Verum tolerated t1
0.004468193	cohens_d	group2	Placebo tolerated t2	Verum complaints t3
0.000192246	cohens_d	group2	Verum tolerated t2	Verum tolerated t3

Table 4: Multivariate effect size calculations by the used metadata categories based on predicted metabolite composition expressed by Bray-Curtis distances.

effect_size	metric	column	group_1	group_2
1.103118308	cohens_d	group2	Placebo tolerated t2	Verum tolerated t2
1.001567582	cohens_d	group2	Placebo tolerated t3	Verum tolerated t2
0.826654574	cohens_d	group2	Placebo tolerated t2	Verum complaints t1
0.796411231	cohens_d	group2	Placebo tolerated t1	Verum tolerated t2
0.770317827	cohens_d	group2	Placebo tolerated t3	Verum complaints t1
0.719520314	cohens_d	group2	Placebo tolerated t2	Verum tolerated t1
0.692224433	cohens_d	group2	Placebo tolerated t3	Verum tolerated t1
0.580587094	cohens_d	group2	Placebo tolerated t3	Verum complaints t3
0.571420685	cohens_d	group2	Placebo tolerated t2	Verum complaints t3
0.569018067	cohens_d	group2	Placebo tolerated t3	Verum complaints t2
0.545091704	cohens_d	group	Placebo	Verum
0.542174844	cohens_d	group2	Placebo tolerated t2	Verum complaints t2
0.539268902	cohens_d	group2	Placebo tolerated t3	Verum tolerated t3
0.520364662	cohens_d	group2	Placebo tolerated t1	Verum complaints t1
0.519543026	cohens_d	group2	Placebo tolerated t2	Verum tolerated t3
0.467334192	cohens_d	group2	Verum complaints t3	Verum tolerated t2
0.449469908	cohens_d	group2	Verum tolerated t2	Verum tolerated t3
0.441447965	cohens_d	group2	Verum complaints t2	Verum tolerated t2
0.430775039	cohens_d	group2	Placebo tolerated t1	Verum tolerated t1
0.35824225	cohens_d	group2	Verum tolerated t1	Verum tolerated t2
0.342271932	cohens_d	group2	Placebo tolerated t1	Placebo tolerated t3
0.300884036	cohens_d	group2	Placebo tolerated t1	Verum complaints t3
0.292836728	cohens_d	group2	Verum complaints t1	Verum tolerated t2
0.291115278	cohens_d	group2	Placebo tolerated t1	Verum complaints t2
0.264439285	cohens_d	group2	Placebo tolerated t1	Verum tolerated t3
0.222359375	cohens_d	group2	Placebo tolerated t1	Placebo tolerated t2
0.218072106	cohens_d	group2	Verum complaints t1	Verum tolerated t3
0.213462164	cohens_d	group2	Verum complaints t1	Verum complaints t3
0.193400394	cohens_d	group2	Verum complaints t1	Verum complaints t2
0.165951089	cohens_d	group2	Placebo tolerated t2	Placebo tolerated t3
0.13813087	cohens_d	group2	Verum tolerated t1	Verum tolerated t3
0.12601857	cohens_d	group2	Verum complaints t3	Verum tolerated t1
0.114286237	cohens_d	group2	Verum complaints t2	Verum tolerated t1
0.084336005	cohens_d	group2	Verum complaints t1	Verum tolerated t1
0.023459173	cohens_d	group2	Verum complaints t2	Verum tolerated t3
0.023170414	cohens_d	group2	Verum complaints t3	Verum tolerated t3
0.001878854	cohens_d	group2	Verum complaints t2	Verum complaints t3

Hence, our comparative power analyses of microbial taxa and predicted metabolic fluxes suggest that a bigger cohort and more samples would have been necessary to determine significance, but our results were rather robust against the selected sub-groupings within our dataset.
